# Experimental study on cement paste using the ultrasonic pulse transmission method

**DOI:** 10.1038/s41597-026-07659-5

**Published:** 2026-07-10

**Authors:** Jakob Harden

**Affiliations:** Graz University of Technology, Faculty of Civil Engineering Sciences, Institute of Technology and Testing of Construction Materials, Graz, Austria

**Keywords:** Civil engineering, Composites, Acoustics

## Abstract

Cement paste is a hydraulic binding agent made of water and cement. It provides the concrete with its solid matrix when hardened. The hardening of the cement paste relies on an exothermic chemical process known as hydration. The progress of hydration and the associated development of mechanical material properties depend primarily on the materials’ composition and hydration temperature. These circumstances were taken into account in the design of this experimental study by varying the water-to-cement ratio and the specimen size. A test series on cement paste made with ordinary Portland cement was conducted using the ultrasonic pulse transmission method with combined compression and shear-wave measurements. Specimens up to 1.5 litres in size were examined. Additionally, density and temperature tests were conducted to describe the specimen’s state before, during, and after the ultrasound tests. All measurement data and metadata were compiled into datasets in the open-source binary format of GNU Octave, published under the Creative Commons Attribution 4.0 International license.

## Background & Summary

Concrete is currently, by far, the most commonly used material for manufacturing building structures. This building material consists of gravel, sand and cement paste. The cement paste is the adhesive that holds the gravel and sand together. It is made of cement and water and undergoes an exothermic chemical reaction called hydration (e.g., Lura *et al*.^1^ The reaction product, the cement stone, provides the concrete its solid material matrix. In technical applications, the development of that chemical reaction is of utmost importance, as it governs the development of cement paste’s mechanical properties.

### Material models

While elastoplastic material models have long been established for hardened concrete (e.g., Li *et al*.^2^; LU *et al*.^3^; SUN *et al*.^4^ they are still under development for the early stage of the hydration process (e.g., Lavergne *et al*.^5^). Designing well-suited and evidence-based material models depends heavily on mechanical test methods, which provide the necessary data. Since the classic test methods used for solids cannot be applied to soft specimens such as cement paste, ultrasound-based test methods have become established in this field.

### Literature

In the relevant literature, neither conclusive mechanical material models nor measurement data are currently available for cement paste in the early hydration stage. For example, the papers of Banouni *et al*.^6^ and Baral & Roesler^7^ are both addressing mechanical properties of cement paste at the early hydration stage using ultrasound-based test methods, but neither of them published the measurement data used for the analysis. The experimental study described here was conducted to address this situation. It provides the required measurement data, thereby paving the way for designing material models for cement paste in the early hydration stage.

### Ultrasound tests

A considerable part of the chemical reaction occurs in the early stages of cement paste hydration. The reaction process depends mainly on the mixing ratio of cement and water and the thermal conditions during the chemical reaction. Therefore, this study focuses on the time and temperature-dependent development of the material properties within the first 24 hours after mixing cement and water (e.g., Maruyama^8^ and Igarashi). The ultrasonic pulse transmission method using piezoelectric contact probes was chosen because it is well suited state-of-the-art technique (e.g., Lim *et al*.^9^) for fully automated and quasi-continuous sound wave measurements of pasty materials.

### Auxiliary tests

**Fig. 1:**
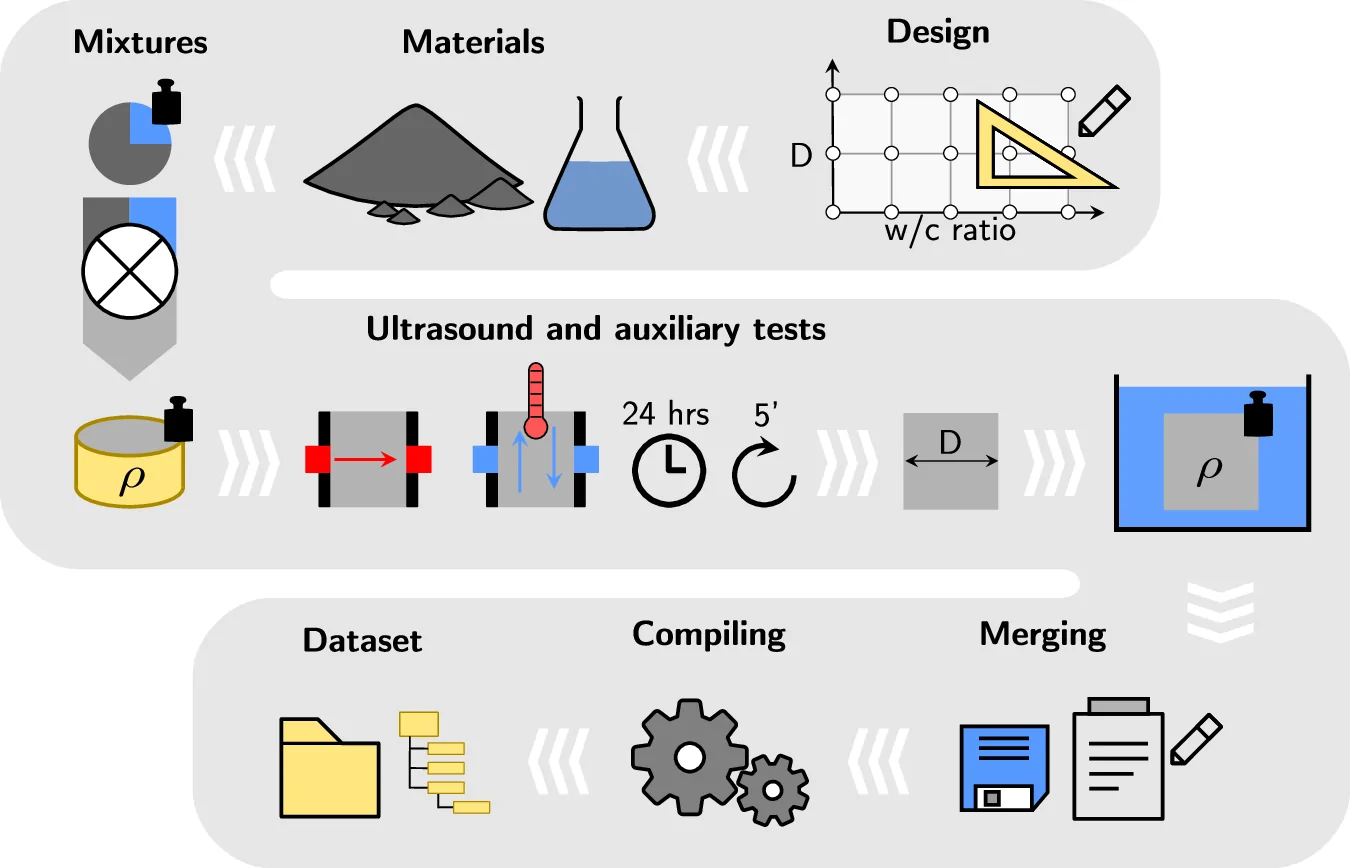
The experimental study – procedural components and workflow.

Besides the ultrasound tests, auxiliary tests were conducted (e.g., specimen temperature, ultrasonic measuring distance and specimen density). These provide further information on the properties of the cement paste specimen before, during and after the ultrasound tests. In Fig. 1, a summary of all procedural components of this experimental study can be found.

### Reference test series

In addition to the test series for cement paste (Test series 1), further reference test series (Test series 5, Test series 6, Test series 7) were conducted using air, water and an aluminium cylinder as testing materials. Since these materials have known material properties, they can serve to investigate the applicability of the ultrasonic test device to different materials and to validate the stability with regard to the reproducibility of the measurement results. Using that reference data for device calibration purposes or accuracy assessments is not intended. This fact is also reflected in the partly roughly estimated ultrasonic measuring distances (see also subsection ‘Testing procedures’ and and section ‘Technical Validation’). The reference test series’ data records are also referred to and described in this work and serve to validate the ultrasound measurements at a technical level. The primary goals of this validation are to assess whether the ultrasound signal recordings reproducibly reflect the fundamental physical principles of sound propagation.

## Data Records

The raw measurement data and metadata from these tests were combined with the ultrasound signal data to form a data set made of text files. The raw data were then compiled into a single binary file. The content of the binary data sets is hierarchically structured, machine-readable and available in the open-source file format of GNU Octave. All data records referenced here are licensed under the Creative Commons Attribution 4.0 International^10^ license and are available for download at the repository^11^ of Graz University of Technology. That permits researchers who do not have access to the necessary experimental equipment, materials or laboratory facilities to benefit from this experimental data. The data records provided can serve as a reference for alternative test setups, materials and analysis methods.

### Data usage

The data records provided are suitable for developing well-suited signal analysis methods and material models, feature extraction and creating training sets for machine learning. In addition, the data can serve as a reference for alternative test setups, materials and analysis methods.

### Application examples

In the past, a subset of the datasets from this study were the basis of the analysis results presented in the form of two conference papers by the author of this study. The first conference paper (see Harden^12^) proposes a method to detect drift signals observed in the ultrasound signal data. The method is based on weighted stochastic properties measured in overlapping signal partitions. The second conference paper (see Harden^13^) concerns signal disturbance characterisation and temporal localisation. The investigated disturbance is the induction current caused by the electromagnetic field emerging from the high-voltage pulse excitation of the piezoelectric activator. Besides the signal’s visual inspection to characterise the disturbance, a matched-filter technique was used for the disturbance’ temporal localisation.

### Scope and content

The scope of this Data Descriptor covers elaborate descriptions about (1) test devices, (2) test procedures and parameters, (3) materials and mixtures, (4) the entire data collection and data processing workflow, and (5) references to the published data.

Not included are (1) predictions for physical material properties, (2) material models, (3) signal analysis results and statistics, (4) discussions, (5) conclusions, and (6) outlook. For technical validation, a minimal analysis is presented to demonstrate the functionality of the ultrasound test device.

## Methods

This section first explains the task, the experimental design and the experimental setup of this study. Then, the materials and the mixtures produced from them are described. Furthermore, the testing devices, the testing device settings, the individual steps of the experiments and the acquisition of the experimental data are comprehensibly detailed. Finally, the methodology of the dataset compilation and the technical validation of the experimental data is presented.

### Study design

The task underlying this experimental study is to create a database. This should serve as a basis for describing the mechanical properties of cement pastes in the early stages of hydration. In order to be able to use the results for technical applications, this study is based on the boundary conditions explained below.

In the early phase of hydration, a substantial part of the exothermic chemical reaction between water and cement occurs. That means that the mechanical properties also change most strongly and most quickly in this phase. The time-dependent evolution of the physical properties of cement paste is discussed by Maruyama^8^ and Igarashi (2014). From those insights, the test duration of 24 hours after adding water to the cement and the measurement interval of five minutes were derived.

For the technically efficient use of cement, it is important to ensure that enough water is added to the mixture to allow the cement to react completely. The optimal amount of water depends mainly on the chemical composition of the cement, the specific weight and the specific surface area of the cement grains. In an experimental study by Wong and Buenfeld^14^, the optimal water-cement ratio (*w*/*c*) for ordinary Portland cement (OPC) was determined to be 0.23. However, for mixtures without superplasticisers a notable higher *w*/*c* ratio in the range of 0.4 and above is used to ensure the paste’s workability. However, the present study focuses on frequently occurring technical applications. Therefore, the study’s lower limit for the *w*/*c* ratio is set to 0.4.

Apart the workability, attention must also be paid to the stability of the mixture. A stable mixture of water and cement should be easy to homogenise and not tend to *bleed*. Bleeding means that some of the water settles again after mixing. This behaviour is often observed at *w*/*c* ratios greater than 0.6. This issue is dealt with in detail in a paper by Ji *et al*.^15^. Based on these findings, the study’s upper limit for the *w*/*c* ratio is set to 0.6.

A geometric boundary condition is given by the ultrasound test apparatus. Before performing the ultrasound tests, the cement paste is loaded into moulds. These consist of two acrylic glass panels with integrated ultrasound contact probes. The vertical cross-sectional area of the interior of the mould, parallel to the acrylic glass panels, is constant. Therefore, the specimen’s volume is determined by the horizontal distance between the two opposite panels. However, this distance can be varied with appropriate spacers and is similar to the distance travelled by the sound waves (ultrasonic measuring distance *D*).

### Study parameters

Based on the properties of the chemical reaction of cement paste, the range of technical applications and the boundary conditions given by the test device, the following essential parameters of this study were identified.

1. **Water-cement ratio**. *w*/*c* = (0.40, 0.45, 0.50, 0.55, 0.60).
2. **Ultrasonic measuring distance**. *D* = (25, 50, 70) mm.
3. **Test duration and measurement interval**. 24 hours with signal and temperature recordings at five-minute intervals.
4. **Repetitions**. Six test runs for each parameter pair (*D*, *w*/*c*).

### Experimental setup

The laboratory tests carried out during this study consist primarily of ultrasonic pulse transmission tests. This testing method is the state-of-the-art technique (e.g., Lim *et al*.^9^) for testing cement paste, cement mortar and concrete in the early stages of hydration. The test device used for the ultrasound tests is the Freshcon^DUO^4.00 device presented by Ruck *et al*.^16^. The following explanations motivate the underlying measuring principle and its technical implementation.

#### Ultrasonic pulse transmission tests

The ultrasound testing device used for this study is the Freshcon^DUO^4.00 ^16^ device. This testing device is designed for fully automated ultrasonic tests on mortar and concrete. Special concern was given to the fact that sound waves in solid and viscoelastic materials propagate not only as longitudinal waves (as in air or water) but also as transversal waves (e.g., Yoshida^17^). For this reason, the Freshcon^DUO^4.00 device has two independent excitation and measurement systems. That experimental apparatus also provides a temperature measuring system which utilises thermocouples (e.g., Roeser^18^) to measure the specimen’s temperature.

**Fig. 2:**
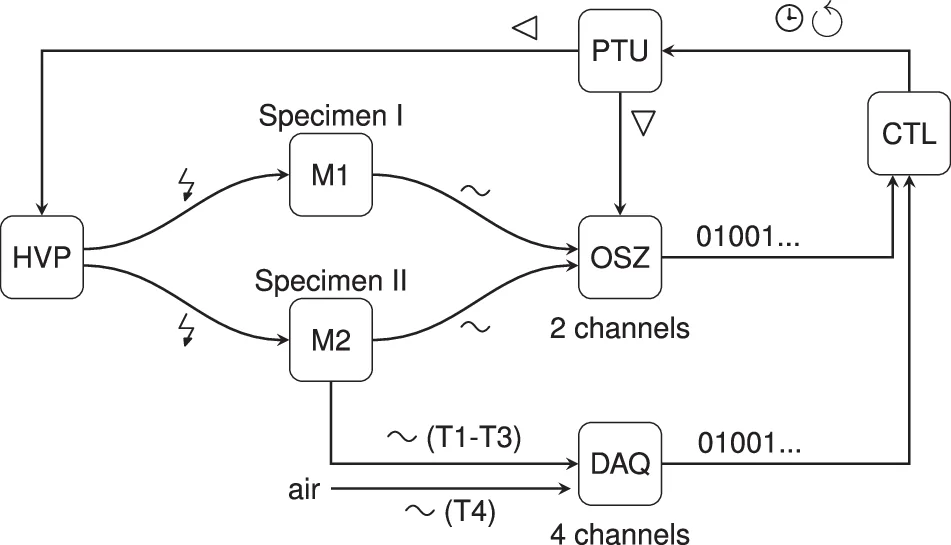
Schematic representation of the ultrasound testing device Freshcon^DUO^4.00. Legend: ↯ …high-voltage pulse; ~ …analogue signal; ⊲ …trigger impulse.

The individual components of the experimental apparatus are detailed in the following list. The corresponding schematic illustration can be found in Fig. 2.

**Fig. 3:**
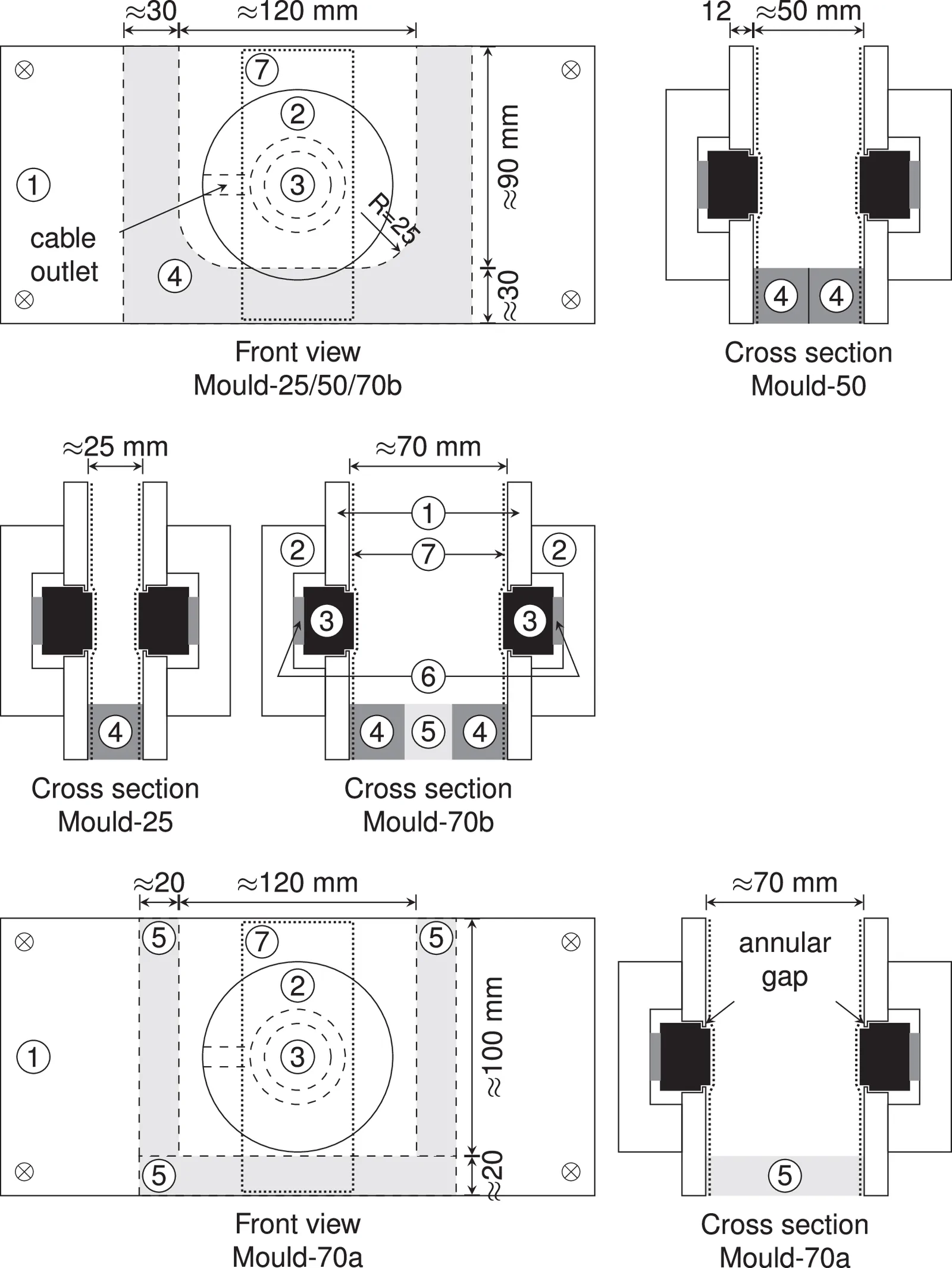
Schematic view of the ultrasound testing device’ mould configuration. Legend: 1… acrylic glass panel (m11), 2… sensor cover (m14), 3… sensor/activator, 4… rubber foam spacer (m12), 5… XPS board spacer (m13), 6… rubber foam backing, 7… polyimide film tape (m7). Note: mould M1 and mould M2 shown in Fig. 2 are, except the built-in ultrasonic contact probes, identically constructed. With respect to the ultrasonic measuring distance *D*, one of the four mould variants (Mould-25, Mould-50, Mould-70a, Mould-70b) was chosen. The material ID’s (e.g., m11) mentioned in the legend refer to the materials listed in Table 6.

**Fig. 4:**
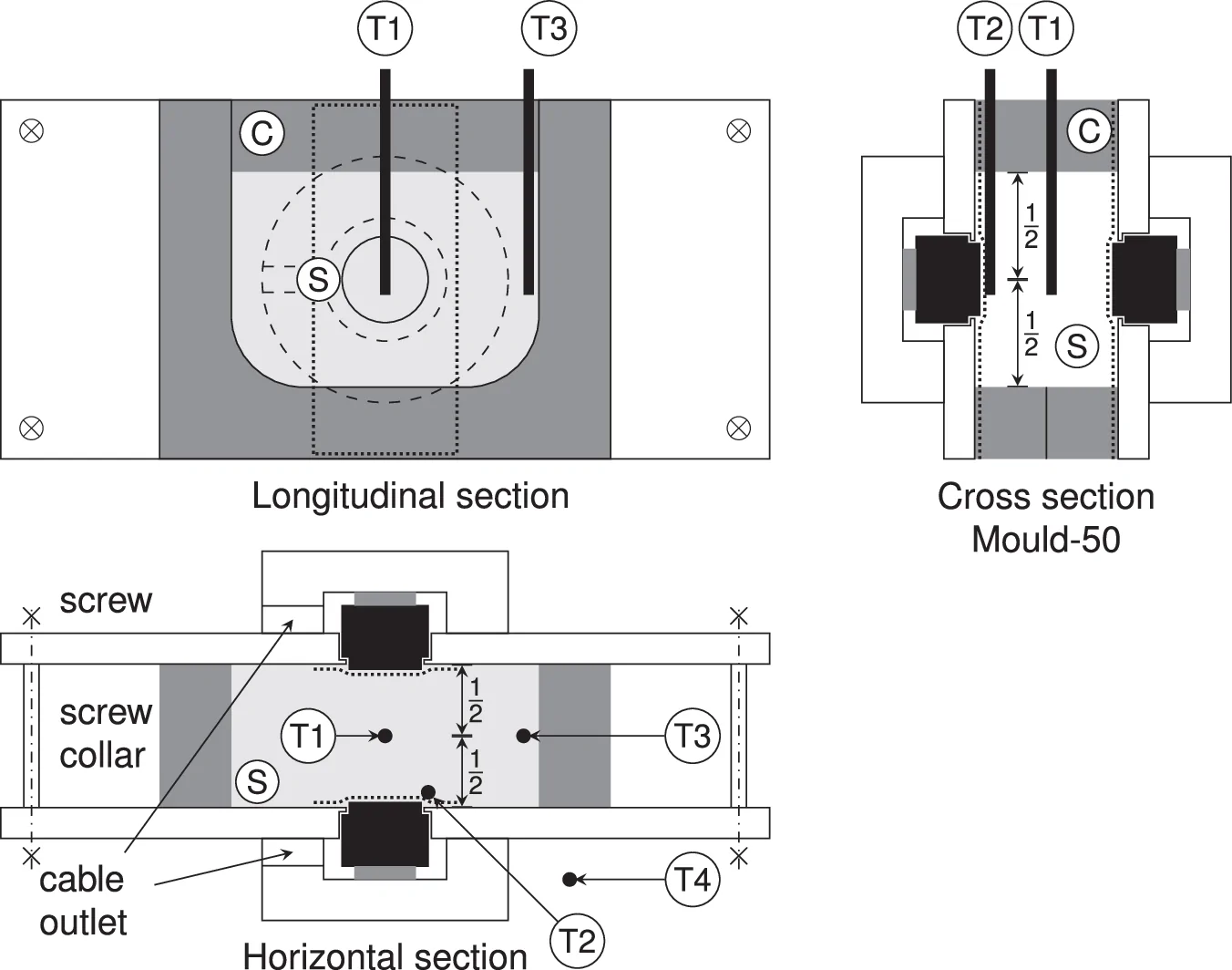
Thermocouple placement schema. Locations inside and outside of mould M2. Legend: S… specimen II, C… cover strips, T1… thermocouple 1, T2… thermocouple 2, T3… thermocouple 3, T4… thermocouple 4.

1. Measuring computer on which the test control and data recording software FreshCon 4.01^19^ is installed (CTL).
2. Pulse trigger unit to synchronise pulse excitation and signal recording (PTU).
3. High-voltage pulse generator for stimulating the activators (HVP).
4. Moulds with built-in activators and sensors (M1, M2). See Fig. 3.
5. Oscilloscope to record the ultrasound signal data (OSZ).
6. Data acquisition unit to record the specimen temperature (DAQ).
7. Thermocouple conductor wires (T1, T2, T3, T4). See Fig. 4.
8. Signal cables (two-wire BNC) connecting PTU, OSZ and HVP.
9. Signal cables (two-wire BNC) connecting HVP with M1 and M2 (pulse).
10. Signal cables (two-wire BNC) connecting OSZ with M1 and M2 (signal).
11. USB cables connecting PTU, OSZ and DAQ with CTL (power supply, data transfer).

#### Auxiliary tests

In addition to the ultrasound tests, auxiliary tests were carried out to determine the environmental conditions and the properties of the specimens before and after the ultrasonic tests. The test equipment used for this purpose as well as for the ultrasound measurements are described in subsection ‘Testing devices’.

### Testing devices

The testing devices listed in Table 1 include the devices used for all laboratory tests during this study. Furthermore, this list is limited to those devices that may influence the measurement results of the ultrasound and the auxiliary tests. Nevertheless, the measuring device specifications are of utmost importance. These were taken from the manufacturer’s data sheets and are summarised in Table 2. To gain more structure, subsets of testing devices were assigned to device groups which are related to distinct testing procedures. The device group definitions, can be found in Table 3.

**Table 1:** Testing devices.

DID	Name	Vendor	Model, Type
d01	Signal cable 1	N/A	BNC, 50 *Ω*/*m*
d02	Signal cable 2	N/A	BNC, 50 *Ω*/*m*
d03	Signal cable 3	N/A	BNC, 50 *Ω*/*m*
d04	Signal cable 4	N/A	BNC, 50 *Ω*/*m*
d09	Thermocouple	RS components	probe type T
d10	Oscilloscope	picoScope	PS-4262
d11	Temperature DAQ	National	cDAQ-9171, NI-9217„
		Instruments	probes: d09
d12	Pulse generator	piezosystems Jena	HVP-1000
d13	Pulse trigger unit	SmartMote	Smart^PULSE^, PTM v1.6
d14	Activator I	Olympus IMS	V101-RB^46^
d15	Sensor I	Olympus IMS	V101-RB^46^
d16	Activator II	Olympus IMS	V150-RB^46^
d17	Sensor II	Olympus IMS	V150-RB^46^
d20	Mold-25	SmartMote	120 × 90 × 25 mm
d21	Mold-50	SmartMote	120 × 90 × 50 mm
d22	Mold-70a	SmartMote	120 × 100 × 70 mm
d23	Mold-70b	SmartMote	120 × 90 × 70 mm
d24	Electronic balance	Sartorius	BP12000S
d25	Electronic balance	Sartorius	LC2200P
d26	Electronic balance	Mettler Toledo	MS16001L
d27	Sliding caliper	Helios-Preisser	DIGI-MET 1320
d28	Compulsory mixer	Hobart	N50-619
d29	Graduated beaker	N/A	volume 1000 cm^3^
d30	Steel washer M12	N/A	DIN 125,
			DIN EN ISO 7089
d31	Thermocouple	RS components	probe type K
d32	Temperature DAQ	Testo GmbH	Testo 177-T4,
			probes: d31
d33	Temperature logger	Testo GmbH	Testo 177-H1
d34	Trowel	N/A	straight edges
d35	Mold-90	SmartMote	120 × 90 × 90 *mm*

Legend: DID …device identifier.

**Table 2:** Measuring device specifications.

DID	Name, Specification	Value, Unit
d10	picoScope PS-4262^47^	—
	vertical, rise time (calculated)	70 ns
	vertical, rise time (±20/ ±10 mV)	88/117 ns
	vertical, resolution	16 bits
	vertical, input sensitivity (10 divisions)	0.002/div÷4/div V
	vertical, input ranges (11 ranges)	±0.01÷±20 V
	vertical, input characteristic, resistance	1 ±0.02 M*Ω*
	vertical, input characteristic, capacity	15 ±2 pF
	vertical, DC acc. (% of full scale)	±0.25%
	vertical, DC acc., (±50/ ±20/ ±10 mV)	±0.5/ ±1/ ±2%
	horizontal, sampling rate	10 MS/s
	horizontal, timebase accuracy	±50 ppm
	horizontal, sample jitter (RMS)	<10 ps
d11	National Instruments cDAQ-9171^48^, NI-9217^49^	—
	vertical resolution	24 bits
	thermocouple probe (Table 1, d09)	type T
	accuracy (25 °C), typical	0.15 °C
	accuracy ( − 40÷+ 70 °C), maximum	0.35 °C
d24	Sartorius BP12000S^50^	—
	resolution (0÷12, 000 g)	0.1 g
	linearity	±0.2 g
	range capability	single (fixed)
d25	Sartorius LC2200P^51^	—
	resolution (0÷2, 200 g)	0.01 g
	linearity (3 ranges)	±0.02/ ±0.02/ ±0.05 g
	range capability	multirange
d26	Mettler Toledo MS16001L^52^	—
	resolution (0÷16, 200 g)	0.1 g
	linearity	±0.2 g
	range capability	single
d27	Helios-Preisser DIGI-MET 1320^53^	—
	resolution (0÷150 mm)	0.01 mm
d32	Testo 177-T4^54^	—
	thermocouple probe (Table 1, d31)	type K
	accuracy (−100÷+ 70 °C)	±0. 3 °C
	resolution (−100÷+ 70 °C)	0.1 °C
d33	Testo 177-H1^55^	—
	accuracy, temperature (−25÷70 °C)	±0. 5 °C
	accuracy, rel. humidity (2÷98%, 25 °C)	±3 ±0.06/Kelvin %
	resolution (temperature, rel. humidity)	±1 digit

Legend: DID …device identifier.

**Table 3:** Device groups.

GID	Device ID’s
g1	d26, d28
g2	d01, d03, d05, d06, d07, d08, d12, d13, d14, d15
g3	d02, d04, d05, d06, d07, d08, d12, d13, d16, d17
g4	d27, d30
g5	d09, d11
g6	d31, d32
g7	d26, d29, d34

Legend: GID …device group identifier.

#### Testing device settings

The testing device settings listed below are limited to those related to the ultrasound tests and thus affect the measuring results. The software settings are also displayed here since the test control and recording software FreshCon 4.01 is an integral part of the ultrasound testing device Freshcon^DUO^4.00. No particular settings had to be made for all other testing devices. All device and software settings are also available from the individual binary data set files described in section ‘Data structure - compiled binary datasets’.

- Oscilloscope (Table 1, d10)
  - Sampling rate: 10 MHz
  - Ensemble averaging: five measurements per sample

- High-voltage pulse generator (Table 1, d12)
  - Pulse voltage: 800 V in Test series 1; 400, 600 and 800 V in Test series 5, Test series 6 and Test series 7.
  - Pulse width: 2.5 *μ*s

- Test control and data recording software FreshCon 4.01
  - Primary channel recording (compression wave signal)
  - Secondary channel recording (shear wave signal)
  - Specimen temperature recording (four channels)
  - Time delay between measurements: five minutes
  - Number of tests *N* = 288 (equal to a test duration of 24 hours)
  - Number of samples recorded before pulse excitation (prefix section length), *N*_pfx_ = 1000 or *N*_pfx_ = 1638 samples

### Materials

In Test series 1, cement pastes were investigated in the early stages of hydration. First, the materials and mixtures used for the cement paste tests are specified. Second, information on the reference materials used in Test series 5, Test series 6, and Test series 7 is provided. Finally, auxiliary materials supporting the testing processes are described.

#### Mixture components

The cement pastes examined were made exclusively from ordinary Portland cement and tap water. The cement was stored on the same laboratory premises where the ultrasound tests were carried out. The tap water comes from the laboratory’s water supply system fed from a tank where the water is kept at room temperature.

The temperature of cement and water was regularly checked with a probe thermometer before mixing them, but was not explicitly recorded in the laboratory protocol. This is because the material temperature never deviated by more than 1 °C from the room temperature. In any case, the measured material temperatures were always within the temperature tolerances specified in subsection subsection ‘Environmental conditions’. Deviations in the signal data related to the lack of exact material temperature measurements are, therefore, at least bounded to some extent. Therefore, the environment temperature measurements provide a helpful indication of the material’s temperature. Nevertheless, it is recommended to measure the materials’ temperature explicitly and record the measurement results in the laboratory protocol.

A brief description of the mixture components and the materials used for the reference tests can be found in Table 4.

**Table 4:** Table of testing materials.

MID	Material	Product	Vendor/Source
m1	Water	tap water	LWSS
m2	Cement	EN 197-1 – CEM I 42.5 N,	VOeZ
		batch 34A	
m3	Cement	EN 197-1 – CEM I 42.5 N,	VOeZ
		batch 35A	
m4	Air	ambient air in laboratory	N/A
m5	Aluminium	cylinder	N/A
		diameter = 48.01 mm	
		height = 49.80 mm	
		weight = 247.8 g	

Legend: MID …material identifier; LWSS …laboratory water supply system; VOeZ …Vereinigung der Österreichischen Zementindustrie.

#### The cement

The cement used in Test series 1 is a CEM I 42,5 N according to the European standard EN 197-1:2011^20^. Furthermore, this cement is a so-called ‘unity cement’ (German: Einheitszement) and is made from a mixture of CEM I 42,5 N cements according to EN 197-1 from all Austrian cement production plants. In the course of Test series 1, cement from two different batches were used. For all mixtures produced up to March 28, 2021, the cement comes from batch 34A. For all subsequent tests, cement from batch 35A was used. The change in batches was not intended but necessary, as not enough cement from batch 34A remained to finish the test series. However, there are several test repetitions available, which can be used to determine whether this change has an impact concerning the test apparatus used to conduct all tests (e.g., ts1_wc040_d25..., ts1_wc040_d50..., ts1_wc045_d25..., ts1_wc045_d50..., ts1_wc050_d25..., ts1_wc050_d50..., ts1_wc050_d70..., ts1_wc055_d25..., ts1_wc055_d50..., ts1_wc055_d70..., ts1_wc060_d25..., ts1_wc060_d50..., ts1_wc060_d70...). See also Tables 7, 9 and 11.

#### Mixture recipes

The mixture recipes for the cement pastes are designed to cover the typical industrial applications. Five mixture compositions with the following water-cement ratios were chosen: *w*/*c* = (0.40, 0.45, 0.50, 0.60). The mass calculations of the mixtures are related to the volume of two moulds of the ultrasound testing device. Since the mould’s volume depends on the designated ultrasonic measuring distances *D* = (25, 50, 70) mm, the mixture compositions shown in Table 5 are broken down according to the water-cement ratio *w*/*c* and the ultrasonic measuring distance *D*.

**Table 5:** Table of mixture compositions.

Mixture		Component 1		Component 2	
XID	*w*/*c*	MID	*m*_*c*_	MID	*m*_*w*_
	[]		[g]		[g]
WC040_D25_B1	0.40	m2	800.0	m1	320.0
WC045_D25_B1	0.45	m2	800.0	m1	360.0
WC050_D25_B1	0.50	m2	800.0	m1	400.0
WC055_D25_B1	0.55	m2	800.0	m1	440.0
WC060_D25_B1	0.60	m2	800.0	m1	480.0
WC040_D50_B1	0.40	m2	1600.0	m1	640.0
WC045_D50_B1	0.45	m2	1600.0	m1	720.0
WC050_D50_B1	0.50	m2	1600.0	m1	800.0
WC055_D50_B1	0.55	m2	1600.0	m1	880.0
WC060_D50_B1	0.60	m2	1600.0	m1	960.0
WC040_D70_B1	0.40	m2	2200.0	m1	880.0
WC045_D70_B1	0.45	m2	2200.0	m1	990.0
WC050_D70_B1	0.50	m2	2200.0	m1	1100.0
WC055_D70_B1	0.55	m2	2200.0	m1	1210.0
WC060_D70_B1	0.60	m2	2200.0	m1	1320.0
WC040_D25_B2	0.40	m3	800.0	m1	320.0
WC045_D25_B2	0.45	m3	800.0	m1	360.0
WC050_D25_B2	0.50	m3	800.0	m1	400.0
WC055_D25_B2	0.55	m3	800.0	m1	440.0
WC060_D25_B2	0.60	m3	800.0	m1	480.0
WC040_D50_B2	0.40	m3	1600.0	m1	640.0
WC045_D50_B2	0.45	m3	1600.0	m1	720.0
WC050_D50_B2	0.50	m3	1600.0	m1	800.0
WC055_D50_B2	0.55	m3	1600.0	m1	880.0
WC060_D50_B2	0.60	m3	1600.0	m1	960.0
WC055_D70_B2	0.55	m2	2200.0	m1	1210.0
WC060_D70_B2	0.60	m2	2200.0	m1	1320.0

Legend: XID …mixture identifier; MID …material identifier (see Table 4); *m*_*c*_ …mass of cement; *m*_*w*_ …mass of water.

#### Reference materials

In addition to Test series 1 on cement pastes, several other ultrasound tests were carried out using materials with known material parameters (air, water, aluminium). These serve as a reference for the cement paste tests and allow for assessing the reproducibility of the ultrasound tests. The reference materials are listed according to the respective test series below.

- Test series 5^21^: ambient air in the laboratory room (see Table 4, m4).
- Test series 6^22^: tap water from the laboratory’s water supply system (see Table 4, m1).
- Test series 7^23^: aluminium cylinder (see Table 4, m5).

#### Auxiliary materials

Additional materials (see Table 6, m6 to m10) were used to prepare the moulds and specimen for the ultrasound tests on cement pastes. The application of these materials is explained in the sampling procedure in subsection ‘Testing procedures’. Apart from that, this list also covers the materials the components of the moulds of the ultrasonic testing device are made of (see Table 6, m11 to m14).

**Table 6:** Table of auxiliary materials.

MID	Description	Material	Vendor
m6	Release agent	Corn germ oil	Mazola
m7	Adhesive tape	Polyimide film tape 5413 width = 50.8 mm thickness = 0.069 mm	3M
m8	Cling film	Polyethylene	N/A
m9	Air balloon	Latex	N/A
m10	Vaseline grease	Paraffin	N/A
m11	Acrylic glass panel	PMMA thickness = 12 mm	N/A
m12	Rubber foam spacer	Cell-caoutchouc, EPDM	N/A
		thickness = 25 mm	
m13	XPS board spacer	Extruded Polystyrene	N/A
		embossed surface	
		thickness = 20 mm	
m14	Sensor cover	Disk, POM	N/A

Legend: MID …material identifier; PMMA …polymethyl methacrylate; EPDM …ethylene propylene diene monomer rubber; POM …polyoxymethylene.

### Environmental conditions

All laboratory tests were carried out in two air-conditioned rooms (Graz University of Technology, Campus Inffeldgasse, Construction Technology Center). Since these rooms are also used for accredited testing, the room temperature and relative humidity are kept within the limits specified below.

- Ambient temperature: *ϑ* = +20 ±1. 5 °C
- Relative humidity: *U* = 60 ±5 %

Whenever these conditions were not met during a test run, this is explicitly noted in the tables next to the device configuration. See also Table 7, Table 9, Table 11, Table 13.

### Testing procedures

**Fig. 5:**
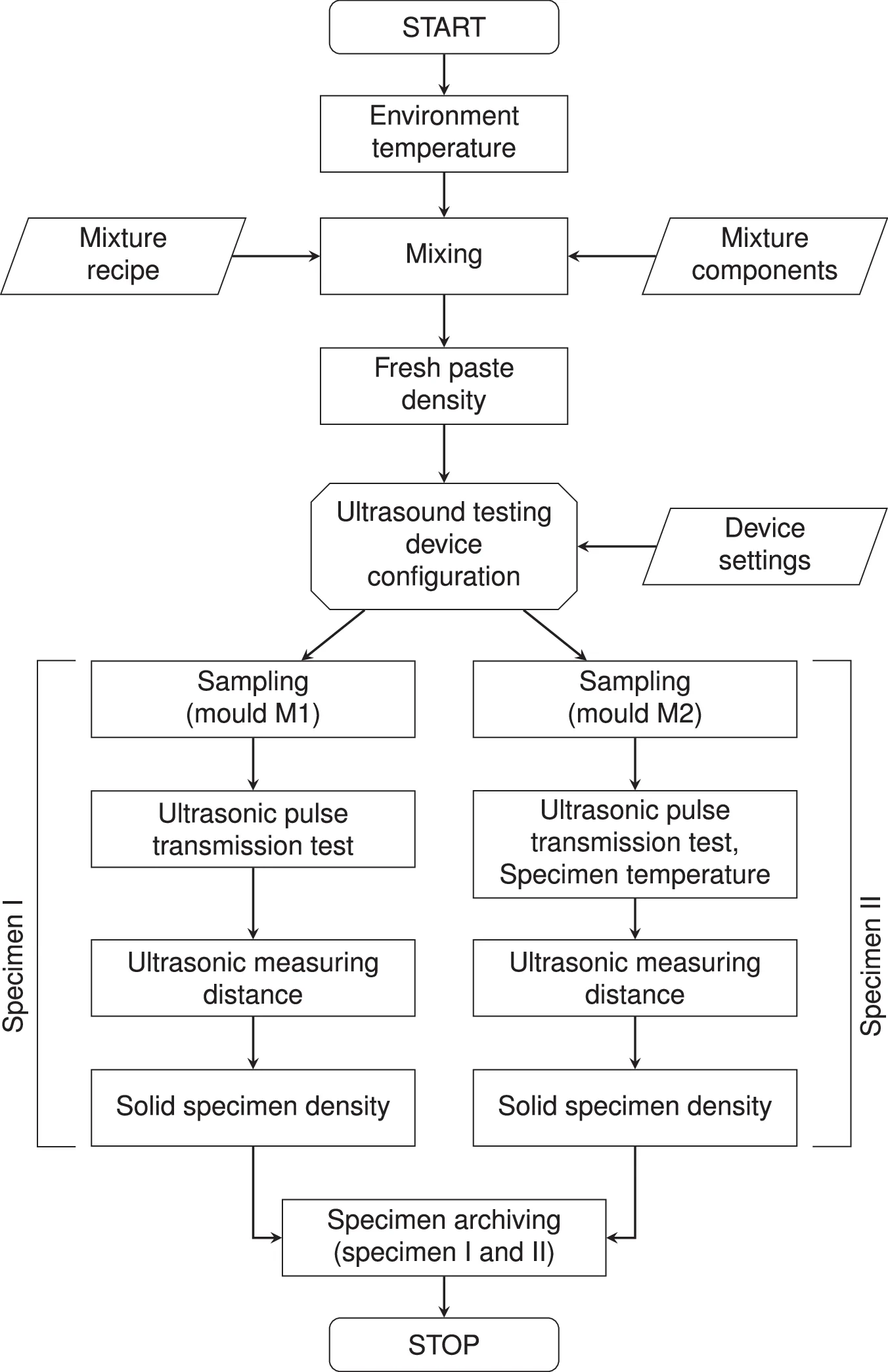
The testing process workflow of a test run in Test series 1.

This section describes the testing procedures of all laboratory tests conducted during this study. Since the laboratory test results of a test run are summarised in the individual datasets of a test series, the relationship between the test series and the laboratory test is established first. Then, the individual laboratory test procedures are explained in detail. The central element of each test run is the ultrasonic pulse transmission test. All other tests provide additional information on the tested specimen and the ambient conditions before, during and after the ultrasound test. Please find a schematic representation of the test sequence of a test run on cement paste in Fig. 5.

The following laboratory tests were carried out for Test series 1. The associated tests and testing equipment can be found in Tables 7-12.

**Table 7:** Testing procedures and test equipment related to the data sets of Test series 1.

Data set	Mixture	Tests, Devices			
designation	XID	AC	ENV	MIX	FPD
ts1_wc040_d25_1	WC040_D25_B1	~	d33	g1	x
ts1_wc040_d25_2	WC040_D25_B1	~	d33	g1	x
ts1_wc040_d25_3	WC040_D25_B1	~	d33	g1	x
ts1_wc040_d25_4	WC040_D25_B1	*✓*	d33	g1	x
ts1_wc040_d25_5	WC040_D25_B1	*✓*	d33	g1	x
ts1_wc040_d25_6	WC040_D25_B2	*✓*	d33	g1	x
ts1_wc040_d50_1	WC040_D50_B1	*✓*	d33	g1	x
ts1_wc040_d50_2	WC040_D50_B1	~	d33	g1	g7
ts1_wc040_d50_3	WC040_D50_B1	~	d33	g1	g7
ts1_wc040_d50_4	WC040_D50_B1	*✓*	d33	g1	x
ts1_wc040_d50_5	WC040_D50_B1	*✓*	d33	g1	g7
ts1_wc040_d50_6	WC040_D50_B2	*✓*	d33	g1	g7
ts1_wc040_d70_1	WC040_D70_B1	~	d33	g1	g7
ts1_wc040_d70_2	WC040_D70_B1	~	d33	g1	g7
ts1_wc040_d70_3	WC040_D70_B1	~	d33	g1	g7
ts1_wc040_d70_4	WC040_D70_B1	*✓*	d33	g1	g7
ts1_wc040_d70_5	WC040_D70_B1	*✓*	d33	g1	g7
ts1_wc040_d70_6	WC040_D70_B1	*✓*	d33	g1	g7
ts1_wc045_d25_1	WC045_D25_B1	~	d33	g1	x
ts1_wc045_d25_2	WC045_D25_B1	*✓*	d33	g1	x
ts1_wc045_d25_3	WC045_D25_B1	*✓*	d33	g1	x
ts1_wc045_d25_4	WC045_D25_B1	*✓*	d33	g1	x
ts1_wc045_d25_5	WC045_D25_B1	*✓*	d33	g1	x
ts1_wc045_d25_6	WC045_D25_B2	*✓*	d33	g1	x
ts1_wc045_d50_1	WC045_D50_B1	~	d33	g1	g7
ts1_wc045_d50_2	WC045_D50_B1	~	d33	g1	g7
ts1_wc045_d50_3	WC045_D50_B1	~	d33	g1	g7
ts1_wc045_d50_4	WC045_D50_B1	*✓*	d33	g1	x
ts1_wc045_d50_5	WC045_D50_B1	*✓*	d33	g1	g7
ts1_wc045_d50_6	WC045_D50_B2	*✓*	d33	g1	g7
ts1_wc045_d70_1	WC045_D70_B1	~	d33	g1	g7
ts1_wc045_d70_2	WC045_D70_B1	~	d33	g1	g7
ts1_wc045_d70_3	WC045_D70_B1	~	d33	g1	g7
ts1_wc045_d70_4	WC045_D70_B1	*✓*	d33	g1	g7
ts1_wc045_d70_5	WC045_D70_B1	*✓*	d33	g1	g7
ts1_wc045_d70_6	WC045_D70_B1	*✓*	d33	g1	g7

Part 1 of 6. Legend: AC …ambient condition status (*✓*: *T* = +20 ±1. 5 °*C*, ~ : conditions deviating from standard conditions); x …test not available.

**Table 8:** Testing procedures and test equipment related to the data sets of Test series 1.

Data set	Tests, Devices					
designation	SMP	UTT^(1)^	UTT^(2)^	TEM	UMD	SSD
ts1_wc040_d25_1	d20	g2	g3	g5	g4	d24
ts1_wc040_d25_2	d20	g2	g3	g5	g4	d25
ts1_wc040_d25_3	d20	g2	g3	g5	g4	d25
ts1_wc040_d25_4	d20	g2	g3	g5	g4	d24
ts1_wc040_d25_5	d20	g2	g3	g5	g4	d24
ts1_wc040_d25_6	d20	g2	g3	g5	g4	d24
ts1_wc040_d50_1	d21	g2	g3	g5	g4	d25
ts1_wc040_d50_2	d21	g2	g3	g5	g4	d25
ts1_wc040_d50_3	d21	g2	g3	g5	g4	d25
ts1_wc040_d50_4	d21	g2	g3	g5	g4	d24
ts1_wc040_d50_5	d21	g2	g3	g5	g4	d24
ts1_wc040_d50_6	d21	g2	g3	g5	g4	d24
ts1_wc040_d70_1	d22	g2	g3	g5	g4	d25
ts1_wc040_d70_2	d22	g2	g3	g5	g4	d25
ts1_wc040_d70_3	d22	g2	g3	g5	g4	d25
ts1_wc040_d70_4	d23	g2	g3	g5	g4	d24
ts1_wc040_d70_5	d23	g2	g3	g5	g4	d24
ts1_wc040_d70_6	d23	g2	g3	g5	g4	d24
ts1_wc045_d25_1	d20	g2	g3	g5	g4	d24
ts1_wc045_d25_2	d20	g2	g3	g5	g4	d24
ts1_wc045_d25_3	d20	g2	g3	g5	g4	d25
ts1_wc045_d25_4	d20	g2	g3	g5	g4	d25
ts1_wc045_d25_5	d20	g2	g3	g5	g4	d24
ts1_wc045_d25_6	d20	g2	g3	g5	g4	d24
ts1_wc045_d50_1	d21	g2	g3	g5	g4	d24
ts1_wc045_d50_2	d21	g2	g3	g5	g4	d24
ts1_wc045_d50_3	d21	g2	g3	g5	g4	d25
ts1_wc045_d50_4	d21	g2	g3	g5	g4	d25
ts1_wc045_d50_5	d21	g2	g3	g5	g4	d24
ts1_wc045_d50_6	d21	g2	g3	g5	g4	d24
ts1_wc045_d70_1	d22	g2	g3	g5	g4	d24
ts1_wc045_d70_2	d22	g2	g3	g5	g4	d24
ts1_wc045_d70_3	d22	g2	g3	g5	g4	d24
ts1_wc045_d70_4	d23	g2	g3	g5	g4	d24
ts1_wc045_d70_5	d23	g2	g3	g5	g4	d24
ts1_wc045_d70_6	d23	g2	g3	g5	g4	d25

Part 2 of 6. Legend: UTT^(1)^, UTT^(2)^ …ultrasonic pulse transmission tests (the superscript denotes the channel number); x …test not available.

**Table 9:** Testing procedures and test equipment related to the data sets of Test series 1.

Data set	Mixture	Tests, Devices			
designation	XID	AC	ENV	MIX	FPD
ts1_wc050_d25_1	WC050_D25_B1	*✓*	d33	g1	x
ts1_wc050_d25_2	WC050_D25_B1	*✓*	d33	g1	x
ts1_wc050_d25_3	WC050_D25_B1	*✓*	d33	g1	x
ts1_wc050_d25_4	WC050_D25_B1	*✓*	d33	g1	x
ts1_wc050_d25_5	WC050_D25_B1	*✓*	d33	g1	x
ts1_wc050_d25_6	WC050_D25_B2	*✓*	d33	g1	x
ts1_wc050_d50_1	WC050_D50_B1	*✓*	d33	g1	g7
ts1_wc050_d50_2	WC050_D50_B1	*✓*	d33	g1	g7
ts1_wc050_d50_3	WC050_D50_B1	*✓*	d33	g1	g7
ts1_wc050_d50_4	WC050_D50_B1	*✓*	d33	g1	g7
ts1_wc050_d50_5	WC050_D50_B1	*✓*	d33	g1	g7
ts1_wc050_d50_6	WC050_D50_B2	*✓*	d33	g1	g7
ts1_wc050_d70_1	WC050_D70_B1	*✓*	d33	g1	g7
ts1_wc050_d70_2	WC050_D70_B1	*✓*	d33	g1	g7
ts1_wc050_d70_3	WC050_D70_B1	*✓*	d33	g1	g7
ts1_wc050_d70_4	WC050_D70_B1	*✓*	d33	g1	g7
ts1_wc050_d70_5	WC050_D70_B1	*✓*	d33	g1	g7
ts1_wc050_d70_6	WC050_D70_B2	*✓*	d33	g1	g7
ts1_wc055_d25_1	WC055_D25_B1	*✓*	d33	g1	x
ts1_wc055_d25_2	WC055_D25_B1	*✓*	d33	g1	x
ts1_wc055_d25_3	WC055_D25_B1	*✓*	d33	g1	x
ts1_wc055_d25_4	WC055_D25_B1	*✓*	d33	g1	x
ts1_wc055_d25_5	WC055_D25_B1	*✓*	d33	g1	x
ts1_wc055_d25_6	WC055_D25_B2	*✓*	d33	g1	x
ts1_wc055_d50_1	WC055_D50_B1	*✓*	d33	g1	x
ts1_wc055_d50_2	WC055_D50_B1	*✓*	d33	g1	x
ts1_wc055_d50_3	WC055_D50_B1	*✓*	d33	g1	x
ts1_wc055_d50_4	WC055_D50_B1	*✓*	d33	g1	g7
ts1_wc055_d50_5	WC055_D50_B1	*✓*	d33	g1	g7
ts1_wc055_d50_6	WC055_D50_B2	*✓*	d33	g1	g7
ts1_wc055_d70_1	WC055_D70_B1	*✓*	d33	g1	x
ts1_wc055_d70_2	WC055_D70_B1	*✓*	d33	g1	x
ts1_wc055_d70_3	WC055_D70_B1	*✓*	d33	g1	x
ts1_wc055_d70_4	WC055_D70_B1	*✓*	d33	g1	g7
ts1_wc055_d70_5	WC055_D70_B1	*✓*	d33	g1	g7
ts1_wc055_d70_6	WC055_D70_B2	*✓*	d33	g1	g7

Part 3 of 6. Legend: see Table 7.

**Table 10:** Testing procedures and test equipment related to the data sets of Test series 1.

Data set	Tests, Devices					
designation	SMP	UTT^(1)^	UTT^(2)^	TEM	UMD	SSD
ts1_wc050_d25_1	d20	g2	g3	x	g4	d24
ts1_wc050_d25_2	d20	g2	g3	x	g4	d24
ts1_wc050_d25_3	d20	g2	g3	x	g4	d24
ts1_wc050_d25_4	d20	g2	g3	g6	g4	d25
ts1_wc050_d25_5	d20	g2	g3	g5	g4	d25
ts1_wc050_d25_6	d20	g2	g3	g5	g4	d24
ts1_wc050_d50_1	d21	g2	g3	x	g4	d24
ts1_wc050_d50_2	d21	g2	g3	x	g4	d24
ts1_wc050_d50_3	d21	g2	g3	x	g4	d24
ts1_wc050_d50_4	d21	g2	g3	g5	g4	d24
ts1_wc050_d50_5	d21	g2	g3	g5	g4	d24
ts1_wc050_d50_6	d21	g2	g3	g5	g4	d24
ts1_wc050_d70_1	d22	g2	g3	x	g4	d24
ts1_wc050_d70_2	d22	g2	g3	x	g4	d24
ts1_wc050_d70_3	d22	g2	g3	x	g4	d25
ts1_wc050_d70_4	d22	g2	g3	g6	g4	d25
ts1_wc050_d70_5	d23	g2	g3	g5	g4	d24
ts1_wc050_d70_6	d23	g2	g3	g5	g4	d24
ts1_wc055_d25_1	d20	g2	g3	x	g4	d24
ts1_wc055_d25_2	d20	g2	g3	x	g4	d24
ts1_wc055_d25_3	d20	g2	g3	x	g4	d24
ts1_wc055_d25_4	d20	g2	g3	g6	g4	d24
ts1_wc055_d25_5	d20	g2	g3	g5	g4	d24
ts1_wc055_d25_6	d20	g2	g3	g5	g4	d24
ts1_wc055_d50_1	d21	g2	g3	x	g4	d24
ts1_wc055_d50_2	d21	g2	g3	x	g4	d24
ts1_wc055_d50_3	d21	g2	g3	x	g4	d24
ts1_wc055_d50_4	d21	g2	g3	g5	g4	d25
ts1_wc055_d50_5	d21	g2	g3	g5	g4	d25
ts1_wc055_d50_6	d21	g2	g3	g5	g4	d24
ts1_wc055_d70_1	d22	g2	g3	x	g4	d24
ts1_wc055_d70_2	d22	g2	g3	x	g4	d24
ts1_wc055_d70_3	d22	g2	g3	x	g4	d24
ts1_wc055_d70_4	d22	g2	g3	g5	g4	d25
ts1_wc055_d70_5	d23	g2	g3	g5	g4	d25
ts1_wc055_d70_6	d23	g2	g3	g5	g4	d24

Part 4 of 6. Legend: see Table 8.

**Table 11:** Testing procedures and test equipment related to the data sets of Test series 1.

Data set	Mixture	Tests, Devices			
designation	XID	AC	ENV	MIX	FPD
ts1_wc060_d25_1	WC060_D25_B1	*✓*	d33	g1	x
ts1_wc060_d25_2	WC060_D25_B1	*✓*	d33	g1	x
ts1_wc060_d25_3	WC060_D25_B1	*✓*	d33	g1	x
ts1_wc060_d25_4	WC060_D25_B1	*✓*	d33	g1	x
ts1_wc060_d25_5	WC060_D25_B1	*✓*	d33	g1	x
ts1_wc060_d25_6	WC060_D25_B2	*✓*	d33	g1	x
ts1_wc060_d50_1	WC060_D50_B1	*✓*	d33	g1	x
ts1_wc060_d50_2	WC060_D50_B1	*✓*	d33	g1	x
ts1_wc060_d50_3	WC060_D50_B1	*✓*	d33	g1	x
ts1_wc060_d50_4	WC060_D50_B1	*✓*	d33	g1	g7
ts1_wc060_d50_5	WC060_D50_B1	*✓*	d33	g1	g7
ts1_wc060_d50_6	WC060_D50_B2	*✓*	d33	g1	g7
ts1_wc060_d70_1	WC060_D70_B1	*✓*	d33	g1	x
ts1_wc060_d70_2	WC060_D70_B1	*✓*	d33	g1	x
ts1_wc060_d70_3	WC060_D70_B1	*✓*	d33	g1	x
ts1_wc060_d70_4	WC060_D70_B1	*✓*	d33	g1	g7
ts1_wc060_d70_5	WC060_D70_B1	*✓*	d33	g1	g7
ts1_wc060_d70_6	WC060_D70_B2	*✓*	d33	g1	g7

Part 5 of 6. Legend: see Table 7.

**Table 12:** Testing procedures and test equipment related to the data sets of Test series 1.

Data set	Tests, Devices					
designation	SMP	UTT^(1)^	UTT^(2)^	TEM	UMD	SSD
ts1_wc060_d25_1	d20	g2	g3	x	g4	d24
ts1_wc060_d25_2	d20	g2	g3	x	g4	d24
ts1_wc060_d25_3	d20	g2	g3	x	g4	d24
ts1_wc060_d25_4	d20	g2	g3	g5	g4	d24
ts1_wc060_d25_5	d20	g2	g3	g5	g4	d25
ts1_wc060_d25_6	d20	g2	g3	g5	g4	d25
ts1_wc060_d50_1	d21	g2	g3	x	g4	d24
ts1_wc060_d50_2	d21	g2	g3	x	g4	d24
ts1_wc060_d50_3	d21	g2	g3	x	g4	d24
ts1_wc060_d50_4	d21	g2	g3	g5	g4	d24
ts1_wc060_d50_5	d21	g2	g3	g5	g4	d25
ts1_wc060_d50_6	d21	g2	g3	g5	g4	d25
ts1_wc060_d70_1	d22	g2	g3	x	g4	d24
ts1_wc060_d70_2	d22	g2	g3	x	g4	d24
ts1_wc060_d70_3	d22	g2	g3	x	g4	d24
ts1_wc060_d70_4	d22	g2	g3	g5	g4	d24
ts1_wc060_d70_5	d23	g2	g3	g5	g4	d25
ts1_wc060_d70_6	d23	g2	g3	g5	g4	d25

Part 6 of 6. Legend: see Table 8.

• Environment temperature (ENV)

• Mixture composing procedure (MIX)

• Fresh paste density (FPD)

• Sampling procedure (SMP)

• Specimen temperature (TEM)

• Ultrasonic pulse transmission tests (UTT)

• Ultrasonic measuring distance (UMD)

• Solid specimen density (SSD)

Due to the nature of the testing material, a workflow different from the one shown above was chosen for the reference tests in Test series 5, Test series 6 and Test series 7. The differences are mainly related to the measurement of the specimen density, temperature, and specimen preparation. The associated tests and testing equipment can be found in Tables 13 and 14.

**Table 13:** Table of testing procedures and test equipment related to the data sets of Test series 5, Test series 6 and Test series 7.

Data set	Material	Tests, Devices					
designation	MID	AC	ENV2	SMP2	SMP3	SMP4	SSD2
ts5_d25...	m4	*✓*	d33	d20	—	—	—
ts5_d50...	m4	*✓*	d33	d21	—	—	—
ts5_d70...	m4	*✓*	d33	d22	—	—	—
ts5_d90...	m4	*✓*	d33	d35	—	—	—
ts6_d25...	m1	*✓*	d33	—	d20	—	—
ts6_d50...	m1	*✓*	d33	—	d21	—	—
ts6_d70...	m1	*✓*	d33	—	d22	—	—
ts6_d90...	m1	*✓*	d33	—	d35	—	—
ts7_d50...	m5	*✓*	d33	—	—	d21	d26

Legend: AC …ambient condition status (*✓*: *ϑ* = +20 ±1. 5 °C and *U* = 60 ±5%, ~ : conditions deviating from standard conditions).

**Table 14:** Table of testing procedures and test equipment related to the data sets of Test series 5, Test series 6 and Test series 7.

Data set	Tests, Devices					
designation	UTT^(1)^	UTT^(2)^	UTT2^(1)^	UTT2^(2)^	UMD2	UMD3
ts5_d25...	g2	g3	—	—	d27	—
ts5_d50...	g2	g3	—	—	d27	—
ts5_d70...	g2	g3	—	—	d27	—
ts5_d90...	g2	g3	—	—	d27	—
ts6_d25...	g2	g3	—	—	d27	—
ts6_d50...	g2	g3	—	—	d27	—
ts6_d70...	g2	g3	—	—	d27	—
ts6_d90...	g2	g3	—	—	d27	—
ts7_d50...	—	—	g2	g3	—	d27

Legend: UTT^(1)^, UTT^(2)^, UTT2^(1)^, UTT2^(2)^ …ultrasonic pulse transmission tests (the superscript denotes the channel number).

• Test series 5, reference tests on air

– Environment temperature and humidity (ENV2)

– Sampling procedure (SMP2)

– Ultrasonic pulse transmission tests (UTT)

– Ultrasonic measuring distance (UMD2)

• Test series 6, reference tests on water

– Environment temperature and humidity (ENV2)

– Sampling procedure (SMP3)

– Ultrasonic pulse transmission tests (UTT)

– Ultrasonic measuring distance (UMD2)

• Test series 7, reference tests on aluminium cylinder

– Environment temperature and humidity (ENV2)

– Sampling procedure (SMP4)

– Ultrasonic pulse transmission tests (UTT2)

– Ultrasonic measuring distance (UMD3)

– Solid specimen density (SSD2)

#### Environment temperature (ENV, ENV2)

Before preparing the mixtures, the environment temperature was read from the room temperature logger and recorded in the laboratory protocol. It is to note that the measured values from the temperature logger and the environment temperature measurement using thermocouples (see testing procedure TEM) differ by approximately 1 °C. For the reference tests in Test series 5, Test series 6 and Test series 7, deviating from the testing procedure ENV, the humidity was also recorded (ENV2).

#### Mixture composing procedure (MIX)

The mixture composing procedure consists of weighing the mixture components and mixing. The individual steps for producing the mixtures are listed below.

1. Mixture components were weighed (digital balance, accuracy ±0.5 g) according to the recipe (see Table 5).
2. Cement was poured into the mixer bowl, the water was added, and the counter-clock was started.
3. The components were mixed for about 60 seconds.
4. Debris was scraped from the mixer bowl wall with a metal spoon, which took about 30 seconds.
5. The mixture was mixed for about 90 seconds.

#### Fresh paste density test (FPD)

The gravimetric determination of the density of the fresh cement paste was carried out directly after preparing the mixtures and before loading the moulds of the ultrasonic testing device. For this purpose, a graduated beaker and a digital balance were used. The weights were measured with an accuracy of ±0.1 g (see Table 2, d26). It should be noted that this test was not carried out in all test runs of Test series 1 and is therefore not available from all datasets. The individual steps of the testing procedure are listed below.

1. The inner surface of the graduated beaker was moistened using a wet towel.
2. Paste was poured into the beaker, and the beaker was filled to approximately half its height.
3. The beaker was shaken for approximately 10 seconds (vertical movements).
4. Paste was poured into the beaker and filled to the top edge.
5. The beaker was shaken for approximately 10 seconds (vertical movements).
6. It was ensured that the beaker was fully filled (plus 2–3 mm over the top edge).
7. Excess material was scraped off along the beaker edge with a trowel.
8. The balance was tared and the gross weight (beaker plus paste) was measured.

#### Sampling procedure (SMP)

The sampling of the test specimens consists of preparing the moulds of the ultrasonic testing device, loading the moulds with the cement paste, compacting the paste and placing the thermocouples for the specimen temperature measurement.

The different mould configurations, please find in Fig. 3. The thermocouple placement is illustrated in Fig. 4. The individual steps of the sampling procedure are listed below.

1. First, it was ensured that the annular gap between the ultrasound contact probes and the acrylic glass panels was properly covered with polyimide film tape.
2. Release agent was applied on the surfaces of mould M1 and mould M2 wetted by the cement paste (corn germ oil, see Table 6, m6).
3. If necessary, the release agent was removed from surface areas of the contact probes using ethanol and a paper towel.
4. Mould M1 and mould M2 were assembled.
5. The cement paste was poured into mould M1 and mould 2 and filled up to approximately half the height of the moulds.
6. Mould M1 and mould M2 were shaken for approximately 10 seconds (vertical movements). Therefore, the mould was lifted on one side by about three to four centimetres and dropped again.
7. The paste was poured into mould M1 and mould M2 and filled up to approximately 2.5 cm below the top edge of the moulds.
8. Mould M1 and mould M2 were shaken for approximately 10 seconds (vertical movements).
9. Mould M1 and mould M2 were covered with cover strips. See note below.
10. Thermocouples were placed as explained in testing procedure TEM (mould M2).

#### Sampling procedure 2 (SMP2)

No special procedures were used to prepare the test specimen for the ultrasound tests in Test series 5. It is worth noting that the ultrasonic contact probes were not covered with polyimide film tape.

#### Sampling procedure 3 (SMP3)

The spacers shown in Fig. 3 are not sufficiently waterproof, the water used for the ultrasound tests in Test series 6 was filled into air balloons (see Table 6, m9), which were subsequently placed between the activator and the sensor of both moulds. The same balloons were used for all ultrasonic measuring distances. For the distance *D* = 90 mm, it was observed that the balloons hardly covered the contact probe’s surface. For this reason, water was added to the balloons, and the tests were repeated. These additional test turns are reflected in the turn number added to the end of the data set designations. This sampling procedure was always carried out without using spacers and polyimide film tape.

#### Sampling procedure 4 (SMP4)

To ensure a flat frictional connection between the aluminium cylinder and the contact probes, a skinny layer of grease (see Table 6, m10) was applied to the contact probe’s surfaces. The aluminium cylinder was then clamped directly between the contact probes, which were not covered with polyimide film tape.

#### Specimen temperature (TEM)

The hydration temperature of the cement paste was measured using thermocouple probes. The temperature was always measured in mould M2 (specimen II). The thermocouple wire’s proper placement can be found in Fig. 4. It has to be noted, that the specimen temperature test was not carried out in all test runs and is therefore not included in all datasets. The individual preparation steps for the temperature measurement are listed below.

1. The insulation of the thermocouples’ ends was stripped, approximately two centimetres long.

2. Both strands were twisted together and bent over, approximately one centimetre long.

3. The first two steps were repeated for all thermocouple wires (T1, T2, T3, T4).

4. Thermocouple T1 was placed in the centre of the specimen in mould M2.

5. Thermocouple T2 was placed on the side panel of mould M2, next to the sensor, vertically centred to the specimen’s height.

6. Thermocouple T3 was placed on the middle of the lateral surface of the spacer of mould M2, vertically centred to the specimen’s height.

7. Thermocouple T4 was placed outside but right next to mould M2.

#### Ultrasonic pulse transmission test (UTT)

Immediately after loading the two moulds and placing the thermocouples, the ultrasonic pulse transmission test was started. The individual steps of the procedure are listed below.

1. The assembly of the testing device (moulds, cables, thermocouple wires) was checked. Thereby, it was ensured that the cables connecting the pulse generator and actuators and those connecting the oscilloscope and sensors were not close to each other. The BNC signal cables used are shielded. However, preceding tests using different cable placements showed that the inductive interference caused by the high-voltage pulse excitation was considerably smaller when the cables were not too close to each other.

2. The software on the measuring computer was started, the test settings were configured (see ‘Testing devices’), and the time elapsed between adding water to the cement and the test initiation was set. See also sampling procedure SMP.

3. The test was initiated and awaited until its end (24 hours).

#### Ultrasonic pulse transmission test 2 (UTT2)

Since only one aluminium cylinder was available, the ultrasound tests in Test series 7 were carried out as single-channel tests. First, all tests were carried out using the longitudinal characteristics activator and sensor in mould M1. Second, all tests were repeated using the transverse characteristics activator and sensor in mould M2. For both test runs, the procedure was the same as described in testing procedure UTT. Finally, the measurement data from both test runs were merged to construct the same data structure as returned by a two-channel test.

#### Ultrasonic measuring distance (UMD)

After removing the hardened cement paste specimen from the moulds, the ultrasonic measuring distance *D* was determined separately for each specimen. Due to the design of the moulds, the ultrasonic contact probes protrude approximately 0.5 mm from the surface of the acrylic glass panels and leave shallow depressions in the hardened test specimens. However, as aid, two steel washers were used to determine the distance (digital sliding caliper, accuracy ±0.01 mm, see Table 2, d27). These were placed in the shallow depressions (hutches) on both sides of the specimen before measuring the distance.

#### Ultrasonic measuring distance 2 (UMD2)

For Test series 5 and Test series 6, the ultrasonic measuring distance *D* was determined by measuring the inner distance between the acrylic glass panels of the moulds (accuracy ±0.01 mm, device d27).

It is to mention, that activator and the sensor are mounted movably in the moulds. Since air does not exert any pressure on the ultrasonic contact probes, they protrude up to 1.5 mm from the surface of the acrylic glass panels. For the reference tests with water, however, a similar situation arises as with cement paste. Due to the water pressure, the probes only protrude about 0.5 mm from the surface of the acrylic glass panels. For this reason, the ultrasonic measuring distance could only be roughly estimated on the basis of the distance between acrylic glass panels and the estimated protrusion.

#### Ultrasonic measuring distance 3 (UMD3)

For the tests in Test series 7, the ultrasonic measuring distance *D* was determined by the distance between the plane-parallel bottom and head surface of the cylinder (accuracy ±0.01 mm, device d27).

#### Solid specimen density (SSD)

The density of the solid test specimens was determined directly after measuring the ultrasonic measuring distance. The density test was done by immersion weighing according to the Archimedean principle (accuracy ±0.1 g, device d25 or accuracy ±0.1 g, device d24, see Table 2). Before the test, loose parts were removed from the specimen’ edges. To prevent the uncoated specimen from absorbing water, the immersion time was kept as short as possible at around 30 seconds.

#### Solid specimen density 2 (SSD2)

In Test series 7 the density of the aluminium cylinder was determined gravimetrically using a digital balance (accuracy ±0.1 g, Table 2, device d27) and a digital sliding caliper (accuracy ±0.01 mm, Table 2, device d26). Due to the accurate geometric dimensions, the volume was calculated from the cylinder dimensions.

### Data acquisition

**Fig. 6:**
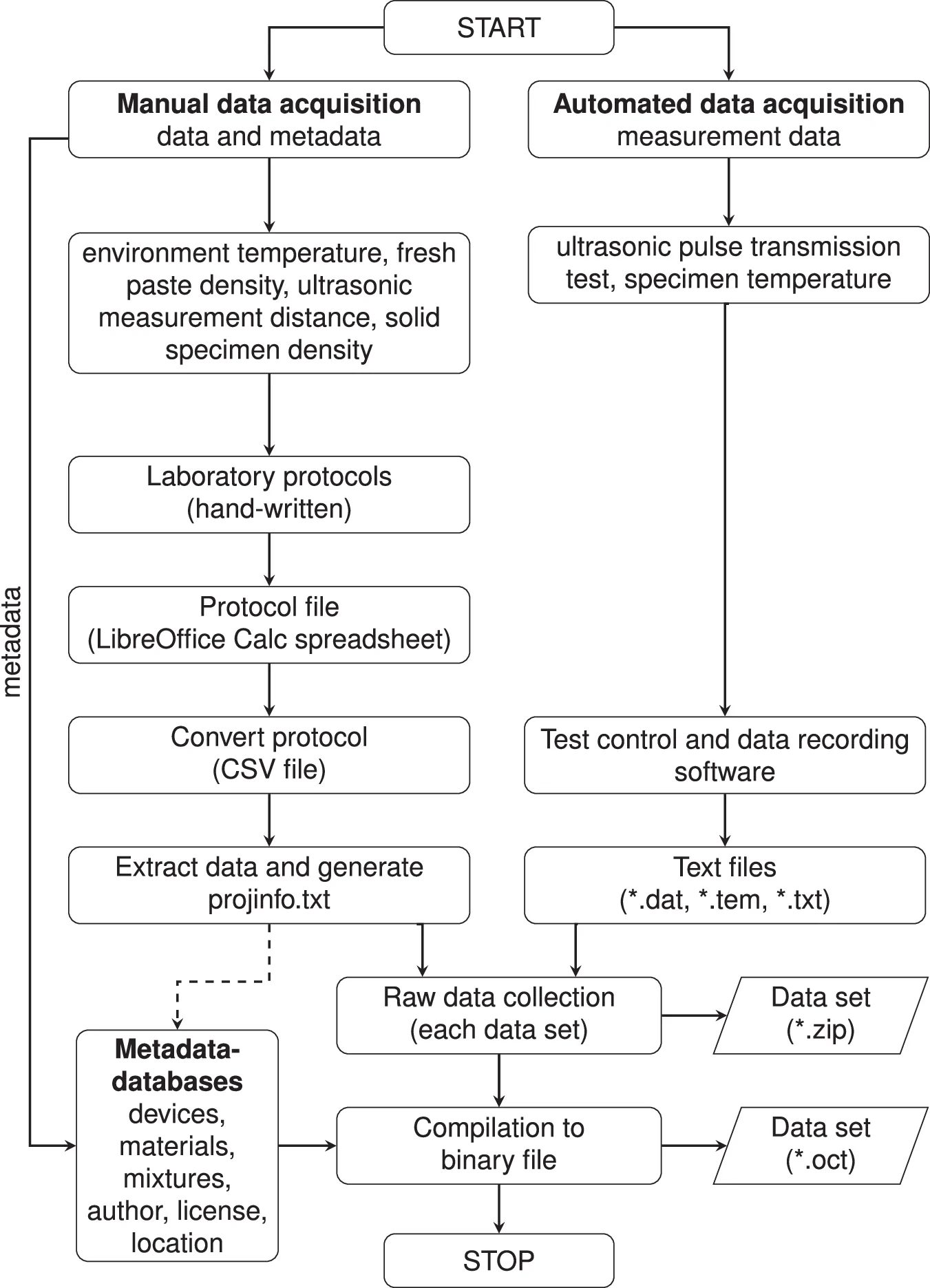
Schematic representation of the data processing workflow. Data acquisition, data collection and data set compilation.

The acquisition of data from the laboratory experiments was carried out either automatically or manually, depending on the type of experiment. A description of the two acquisition methods is given below. In Fig. 6, please find a schematic representation of the data collection and processing workflow.

#### Automated data acquisition

The signal data of the ultrasound tests (UTT, UTT2) and the specimen temperature (TEM) were recorded automatically by the test control and data recording software FreshCon 4.01. The software reads the digitised data from the data acquisition units (see Table 1, device d10, d11) at specified measurement intervals over the entire test duration and stores them in plain text files.

After the ultrasound test, the measurement data are available as human- and machine-readable text files. The respective folder and file structure of the recordings is explained in subsection ‘Data structure - raw data datasets’.

One aspect of automated data acquisition using the Freshcon^DUO^4.00 device^16^ should be emphasised in particular. The USB oscilloscope (see Table 1, device d10), which is part of that device, has a built-in automatic amplitude pre-amplification with eleven voltage ranges (see Table 2, device d10). Therefore, the signal responses of the compression and shear waves are scaled to some extent. The actual pre-amplification level of the oscilloscope is displayed on the screen but not recorded by the software and therefore not available. This aspect is also mentioned in the device’s user manual^19^ on page 25. The pre-amplifications’ effect becomes particularly apparent in the graphical representations of the analysis results shown in section ‘Technical Validation’.

#### Manual data acquisition

The measurement data of all other laboratory tests (ENV, ENV2, FPD, UMD, UMD2, UMD3, SSD, SSD2) described in section ‘Testing procedures’ were recorded manually in handwritten laboratory protocols. The data were then transferred to a LibreOffice^24^ Calc spreadsheet and exported as a comma-separated text file. These text files are the basis for the file projinfo.txt described in subsection ‘Data structure - raw data datasets’. Subsequently, this file was assigned to the raw data folders of the individual datasets.

For the metadata (e.g., information on test equipment, materials, mixtures, procedures), metadata databases were created using the script collection ‘Data set compiler 1.2’^25^ and the open-source binary file format of GNU Octave. These databases provide the metadata for the binary datasets compiled from the collected raw data. They are an integral part of the script collection ‘Data set compiler 1.2’ described in section ‘Code Availability’ The databases and the GNU Octave function files used to generate them are located in the script collection’s folder./struct_hdf/db/^25^.

It is worth mentioning that the projinfo.txt file contains only the record identification numbers from the metadata database instead of the complete metadata. Based on those identification numbers, metadata from the afore-mentioned databases is inserted into the binary datasets during the fully automated compilation process.

### Computational processing

The computational processing of the measurement data and the associated metadata refers exclusively to the fully automated compilation of the collected raw data into binary datasets. The measurement data were neither filtered nor modified in any other way. An illustration of this process, please find in Fig. 6. The individual steps of the compilation process are listed below.

• Read in the measurement data from the automated data acquisition (tst<num>.dat, tst.tem, measurements.txt, settings.txt).

• Read in the measurement data from the manual data acquisition (projinfo.txt).

• Assembled metadata structure using the file projinfo.txt and the metadata databases.

• Assembled data structure for the individual laboratory experiments from the measurement data.

• Assembled dataset structure using the already assembled data and metadata substructures.

• Saved the assembled data set structure in a data set file using the binary file format of GNU Octave.

The data processing steps are only roughly described here. To make the whole process comprehensible, the script collection Data set compiler 1.2^25^ used for this task is also made available and described in section ‘Code Availability’. The inclined reader may want to scrutinise the program code to comprehend all details of the compilation process (start with file ./struct_hdf/import/struct_import.m).

### Analysis methods for the technical validation

The analysis methods described here are used to justify the technical validity of the ultrasound measurement results. The validation aims to show that the recorded ultrasound signal data reproducibly reflects the underlying physical laws of sound propagation. The following explanations describe the selected materials (datasets), the analysis methods and the analysis procedure.

#### Materials (datasets)

To cover the broadest possible range of materials, a selection of data sets from the aforementioned reference tests with air, water and an aluminium cylinder are used. These materials cover all three aggregation states possible under normal ambient conditions. Since the physical properties of the reference materials are known from the literature, an independent prediction of the expected material behaviour concerning the given test conditions is possible. In addition to the material’s aggregate state, the ultrasonic measuring distance also plays an important role in the propagation of the sound waves. This circumstance is reflected by using signal data from ultrasound tests with various measuring distances. The following list retains the selection of the data sets subjected to the analysis.

- Test series 5, reference tests on air^21^

— *D* = 25 mm: ts5_d25_b16_v800.oct, ts5_d25_b24_v800.oct, ts5_d25_b33_v800.oct, ts5_d25_b50_v800.oct

— *D* = 50 mm: ts5_d25_b16_v800.oct, ts5_d25_b24_v800.oct, ts5_d25_b33_v800.oct, ts5_d25_b50_v800.oct

— *D* = 70 mm: ts5_d25_b16_v800.oct, ts5_d25_b24_v800.oct, ts5_d25_b33_v800.oct, ts5_d25_b50_v800.oct

- Test series 6, reference tests on water^22^

— *D* = 25 mm: ts6_d25_b16_v800.oct, ts6_d25_b24_v800.oct, ts6_d25_b33_v800.oct, ts6_d25_b50_v800.oct

— *D* = 50 mm: ts6_d25_b16_v800.oct, ts6_d25_b24_v800.oct, ts6_d25_b33_v800.oct, ts6_d25_b50_v800.oct

— *D* = 70 mm: ts6_d25_b16_v800.oct, ts6_d25_b24_v800.oct, ts6_d25_b33_v800.oct, ts6_d25_b50_v800.oct

- Test series 7, reference tests on aluminium cylinder^23^

—*D* = 50 mm: ts7_d25_b16_v800.oct, ts7_d25_b24_v800.oct, ts7_d25_b33_v800.oct, ts7_d25_b50_v800.oct

Each data set contains ten recordings of the signal responses of compression and shear waves. For air and water, however, only the signal responses of the compression wave are considered. For the aluminium cylinder, the signal responses of the shear waves are also considered since solid materials also transmit transverse waves.

#### Analysis methods

To show that the ultrasound tests’ signal responses reflect the underlying physical laws of sound propagation, a prediction of the sound travel time is compared with compression and shear wave signal responses. In addition, the reproducibility of the tests is justified using the stochastic properties of signal sequences of repeated tests. The analysis methods used are briefly summarised in the following list.

- Estimating the sound travel time based on the ultrasound measurement distance and the literature data for the reference materials’ speed of sound. The ambient conditions (temperature, humidity), the measurement tolerances of the test equipment and procedural errors are taken into account in the estimation. Furthermore, it is assumed that the measured values and the associated tolerances are not correlated.
- Combining signal data from repeated tests to signal sequences. The ensemble average and the distribution of the deviations of the signal amplitudes from the ensemble average are determined from the signal sequences, as these reflect the average behaviour of the ultrasound test configuration more reliably. Minimum, maximum and quantiles characterise the distribution of the deviations.
- Visual comparison of the sound travel times of the first incoming wave and its reflections with the tolerance ranges of the predicted sound travel times.
- Visual comparison of the ensemble averages amplitudes of the first incoming wave and its reflections.
- Assessment of the reproducibility based on the characteristic properties of the deviations’ distribution.

#### Analysis procedure

The subsequently described analysis procedure is derived from the task of technical validation and the chosen materials and methods. The detailed explanations do not aim to make the most accurate prediction possible but rather to consider all known boundary conditions.

- Estimation of the ultrasonic measuring distance limits $\widehat{D}$ considering the measured distance *D*, procedural errors and the measuring device tolerances (see Table 2, device d27). The source of the considerably high procedural errors are described in detail in the testing procedures UMD2 and UMD3 in subsection ‘Testing procedures’.

— Air: $$\widehat{D}=D-3/+0\pm 0.01$$ mm

— Water: $$\widehat{D}=D-1/+0\pm 0.01$$ mm

— Aluminium cylinder: $\widehat{d}=d\pm 0.01$ mm

- Estimation of temperature limits $\widehat{{\vartheta }}$ for the materials air and water considering the measured ambient temperature *ϑ* and the measuring device tolerances (see Table 2, device d33). $\widehat{{\vartheta }}={\vartheta }\pm 0.{3}^{\circ }$C.
- Estimation of the humidity limits $\widehat{U}$ for air considering the measured ambient humidity *U* and the measuring device tolerances (see Table 2, device d33). It is to be mentioned that the temperature-dependent measuring tolerance estimate (% per Kelvin) is based on the difference between the ambient temperature *ϑ* and the device calibration standard temperature (*ϑ*_std_ = 25 °C). $\hat{U}=U\pm 3\pm (0.06\cdot |25-{\vartheta }|)$%.
- Estimation of the aluminium cylinder’s density limits $\widehat{\rho }$ considering the cylinder dimensions, its weight and the measuring device tolerances (see Table 2, devices d26, d27).

— Diameter: $$\widehat{d}=d\pm 0.01$$ mm

— Height: $$\widehat{h}=h\pm 0.01$$ mm

— Volume: $$\widehat{v}={\widehat{d}}^{2}\,\frac{\pi \,\widehat{h}}{4\cdot 1000}$$ cm^3^

— Mass: $$\widehat{m}=m\pm 0.1$$ g

— Density: $$\widehat{\rho }=\frac{\widehat{m}}{\widehat{v}}$$ g/cm^3^

- Estimation of the speed of sound limits $\widehat{c}$ based on literature values and the previously estimated limits for temperature $\widehat{{\vartheta }}$ and relative humidity $\widehat{U}$. The speed of sound for air^26^ and water^27^ were interpolated linearly using the tabulated values from the literature. As the elastic properties of the aluminium cylinder were not explicitly measured, and no other more precise information on the actual material is available, the speed of sound was calculated (e.g., Garett^28^) from the cylinder’s density and the elastic moduli taken from literature^29^. The tolerance limits for the Young’s modulus *E* and the shear modulus *G* refer to the tabulated values of the aluminium alloys 1100 and 2014.

— Air: $\widehat{c}={I}_{2}(\widehat{{\vartheta }},\widehat{U})$ m/s, where *I*_2_ is a two-dimensional linear interpolation with the supports $\widehat{{\vartheta }}$ and $\widehat{U}$ realised by Octave’s built-in function *interp1*.

— Water: $\widehat{c}={I}_{1}(\widehat{{\vartheta }})$ m/s, where *I*_1_ is a one-dimensional linear interpolation with the support $\widehat{{\vartheta }}$ realised by Octave’s built-in function *interp2*.

— Aluminium:

* Poisson’s ratio ν for isotropic elastic materials. See equation 1.

* Speed of sound c of the compression (longitudinal) wave in isotropic elastic materials. See equation 2.

* Speed of sound c of the shear (transverse) wave in isotropic elastic materials. See equation 3.

- Estimation of the signal index ${\widehat{n}}_{0}$ at which the high-voltage pulse excitation apparently happened. A short period (trigger delay) passes between the zero point of the samples’ time signatures and the beginning of the inductive disturbance caused by the high-voltage pulse. The time period is too long to be explained by the propagation speed of the electromagnetic wave. The fact that several processor cycles are required to process the trigger event is a likely cause. This delay is estimated based on visual observations and existing analyses^13^ with ${\hat{n}}_{0}=10\,+5/-2$ samples and added to the sound travel time estimates.
- Estimation of the sound travel time tolerance ranges ${\widehat{t}}_{1}$, ${\widehat{t}}_{2}$, ${\widehat{t}}_{3}$ from the trigger delay limits ${\widehat{n}}_{0}$, the ultrasonic measuring distance limits $\widehat{D}$ and the speed of sound limits $\widehat{c}$ for the first incoming sound wave and the first and second reflection of the sound wave.

– Zero-time (trigger delay): ${\hat{t}}_{0}=\frac{{\hat{n}}_{0}}{{f}_{s}}\cdot 1{0}^{6}\,\mu {\rm{s}}$, sampling rate *f*_*s*_ = 10 MHz (see testing device settings in subsection ‘Testing devices’)

– Sound travel time of the first incoming wave: ${\widehat{t}}_{1}=(\frac{\widehat{c}}{\widehat{D}}+{\widehat{t}}_{0})\cdot 1{0}^{6}\,\mu {\rm{s}}$

– Sound travel time of the wave’ first reflection: ${\widehat{t}}_{2}=(3\,\frac{\widehat{c}}{\widehat{D}}+{\widehat{t}}_{0})\,\mu {\rm{s}}$

– Sound travel time of the wave’ second reflection: ${\widehat{t}}_{3}=(5\,\frac{\widehat{c}}{\widehat{D}}+{\widehat{t}}_{0})\,\mu {\rm{s}}$

- Combining signals from different data sets of test repetitions into signal sequences. This results in a signal sequence (*x*_*k*_) including *N*_*k*_ = 40 signals per distance and material.
- Calculation of the ensemble average *M* and the distribution of the deviations *R* from the ensemble average. The distribution of *R* is thereby characterised by the minimum ${R}_{\min }$, the maximum ${R}_{\max }$, the lower quartile *R*_*Q*(0.25)_ and the upper quartile *R*_*Q*(0.75)_. It is to be noted that the quartile estimates are realised using Octave’s built-in function *quantile* (method No. 5).

– Ensemble average M[n]. See equation 4.

– Deviation *R*[*k*, *n*] from the esemble average of individual signals. See equation 5.

- Plotting the analysis results.
- Visual comparison of the estimated sound travel times ${\widehat{t}}_{x}$ with the ensemble average *M*.
- Visual inspection of the distribution *R* of the deviation from the ensemble average *M*.

1 $$\widehat{\nu }=\frac{\widehat{E}}{2\,\widehat{G}}-1$$

2 $$\widehat{c}=\sqrt{\frac{\widehat{E}(1-\widehat{\nu })}{\widehat{\rho }(1+\widehat{\nu })(1-2\,\widehat{\nu })}\cdot 1{0}^{6}}\,{\rm{m/s}}$$

3 $$\hat{c}=\sqrt{\frac{\hat{G}}{\hat{\rho }}\cdot 1{0}^{6}}\,{\rm{m}}/{\rm{s}}$$

4 $$M[n]=\frac{1}{{N}_{k}}{\sum }_{k=0}^{{N}_{k}-1}{x}_{k}[n]$$

5 $$R[k,n]={x}_{k}[n]-M[n],\ 0\le k\le {N}_{k}$$

The realisation of the analysis procedure (GNU Octave code) is provided for comprehensibility and reproducibility reasons and referred to in section ‘Code Availability’. The instructions for reproducing the analysis results are provided in section ‘Usage Notes’. The results of the above–described analysis are presented in section ‘Technical Validation’.

## Data Records

This section is dedicated to describing the content and structure of all data records referenced in this work. These include the datasets from the experimental study on cement pastes and the datasets from the reference tests with air, water and the aluminium cylinder.

The following subsections explain availability, file naming conventions, file formats, content and the structure of the datasets included in the data records in detail.

### Availability

All data records are made available as digital objects at the *TU Graz Repository*^11^ –. To ensure sustained usability, the they are licensed under the Creative Commons Attribution 4.0 International (CC BY 4.0) license^10^. The following list concludes the data records associated with this study.

- Test series 1, cement paste tests^30^.
- Test series 5, reference tests on air^21^.
- Test series 6, reference tests on water^22^.
- Test series 7, reference tests on aluminium cylinder^23^.

### Record content

Each data record includes a compressed TAR archive containing the raw data of the laboratory experiments, a compressed TAR archive with the datasets compiled from them and a technical description in the form of a PDF file. The archive structure of each record is shown below. The ⟨ts⟩ prefix denotes the actual test series code, and the asterisk is a placeholder for the unique data set identifiers also referred here to as the data set designation.



#### Access to the record content

Each of the aforementioned TAR archives contains the datasets of an entire test series. Therefore, the TAR archives must first be decompressed and unpacked. A description of the procedure and the required software is available in section ‘Usage Notes’.

#### Dataset designation and filename conventions

The datasets’ file names reflect the variation of each test series’ test parameters. The file names – without considering the filename extension – represent the dataset designation. A meaningful file name makes searching for individual datasets easier, and its uniqueness enables clear referencing. Since the individual test series rely on different parameter variations, different conventions apply to the data set’s file names. The following listings provide an overview of the filename conventions of the respective test series. How to read them, is explained by examples.

**Test series 1 (90 datasets)**

Filename pattern := ⟨ts⟩_wc⟨W⟩_d⟨D⟩_⟨R⟩.⟨E⟩

⟨*ts*⟩ test series identifier ts1

⟨*W*⟩ water-cement ratio *w*/*c* (0.40, 0.45, 0.50, 0.55, 0.60) × 100

⟨*D*⟩ distance *D* (25, 50, 70) mm

⟨*R*⟩ repetition, turn (1,…,6)

⟨*E*⟩ filename extension oct, zip

**Example:** ts1_wc055_d70_1.oct is a binary data set of test series 1. The water-cement ratio *w*/*c* of the cement paste is 0.55, the ultrasonic measuring distance *D* is 70 mm, and the turn or repetition number is 1. The filename of the corresponding raw data archive is ts1_wc055_d70_1.zip.

**Test series 5 (48 datasets)**

Filename pattern := ⟨ts⟩_d⟨D⟩_b⟨L⟩_v⟨V⟩.⟨E⟩

⟨*ts*⟩ test series identifier ts5

⟨*D*⟩ distance *D* (25, 50, 70, 90) mm

⟨*L*⟩ recording length (16, 24, 33, 50) kilo-samples

⟨*V*⟩ pulse voltage (400, 600, 800) Volts

⟨*E*⟩ filename extension oct, zip

**Example:** ts5_d25_b50_v800.oct is a binary data set of test series 5. The ultrasonic measuring distance *D* is 25 mm, the recording length (block size) *L* is 50 kilo-samples, and the excitation pulse voltage *V* is 800 Volts. The file name of the corresponding raw data archive is ts5_d25_b50_v800.zip.

**Test series 6 (60 datasets)**

Filename pattern := ⟨ts⟩_d⟨D⟩_b⟨L⟩_v⟨V⟩_[⟨R⟩].⟨E⟩

⟨*ts*⟩ test series identifier ts6

⟨*D*⟩ distance *D* (25, 50, 70, 90) mm

⟨*L*⟩ recording length (16, 24, 33, 50) kilo-samples

⟨*V*⟩ pulse voltage (400, 600, 800) Volts

⟨*R*⟩ repetition, turn (1,2)

⟨*E*⟩ filename extension oct, zip

Please note that the repetition number ⟨R⟩ is available for the test runs of Test series 6 with *D* = 90 mm only. This circumstance is explained in the sampling procedure SMP3 in section ‘Testing procedures’.

**Test series 7 (12 datasets)**

Filename pattern := ⟨ts⟩_d⟨D⟩_b⟨L⟩_v⟨V⟩.⟨E⟩

⟨*ts*⟩ distance *D* 50 mm

⟨*L*⟩ recording length (16, 24, 33, 50) kilo-samples

⟨*V*⟩ pulse voltage (400, 600, 800) Volts

⟨*E*⟩ filename extension oct, zip

#### File formats

For all files in the data records only open-source file formats are used. The file formats and the software used to create these files are listed below.

**Extension** ... **Description** / **Software**

**.tar.xz** ... compressed TAR archive / GNU tar 1.34^31^

**.zip** ... ZIP archive / Gzip 1.10^32^

**.oct** ... Octave binary file / GNU Octave 6.2.0^33^

**.dat, .tem, .txt** ... plain text, encoding us-ascii / FreshCon 4.01^19^

### Data structure – raw data datasets

The raw data archives (…_rawdata.tar.xz) contain a set of ZIP archives (.zip). These consist of the collected raw data from all experiments related to one test run. The raw data are human- and machine-readable text files whose contents have different structures. For all text files, comment lines always begin with two pond signs (##) which are silently ignored during machine interpretation. The tree view below shows the directory and file structure in the raw data ZIP archives.



#### File structure – projinfo.txt

The projinfo.txt file supplies all manually collected laboratory test results. In addition, this file contains the metadata about the test series and the tests. Each line of this file represents an independent entry with the following line format: [datatype] tag = value. The data type descriptor [datatype] in brackets can take one of the following values: (1) str for character arrays; (2) bool for Boolean values (true or false); (3) uint for unsigned integral numbers; (4) sng for single precision floating point numbers (32 Bit); (5) dbl for double precision floating point numbers (64 Bit). Boolean values and character arrays (strings) are always surrounded by double-quotes (e.g., "string", "true").

#### File structure – measurements.txt

The measurements.txt file is a tab-separated text file. It contains a two-column list with file names (e.g., tst0001.dat) and the signal recording times (e.g., 01:26:30). The times refer to the time when the automated test execution was initiated (t = 0 s).

#### File structure – settings.txt

The settings.txt file is a tab-separated text file. It contains the software settings used to control the automated execution of the ultrasonic pulse transmission tests. The first column holds the names of the settings; the second column retains the corresponding values.

#### File structure – tst.tem

The tst.tem file is a tab-separated text file and contains the recordings of the automated temperature measurement of the test specimens. The first line consists of the name of the recording software and the second line the channel identification numbers of the four available channels. In all subsequent lines the temperature recordings of each channel (columns 1 to 4, °C) and the recording time (column 5, seconds) are stored. The recording time refers to the time when the automated test execution was initiated (t = 0 s).

#### File structure – tst⟨num⟩.dat

The tst⟨num⟩.dat files are tab-separated text files and contain the automated ultrasound signal data recordings where each line represents the time-amplitude tuple of one sample. The first column holds the sample time signatures in seconds, and the second column holds the signal amplitudes in volts. The numerical values in both columns are signed and limited to eleven digits. The trigger point (the point in time when the high-voltage pulse was triggered) can be identified by the time signature 0.0000000000. The time signatures before the trigger point have a negative sign and represent the signal’s preamble (prefix or pre-trigger section). The signal amplitudes in the signal’s preamble consist of noise floor only.

### Data structure – compiled binary datasets

The data set archives (…_dataset.tar.xz) contain a collection of binary files (.oct). These binary datasets have a hierarchical data structure that resembles a directory structure. It is made of a basic structure with substructures and data elements. All structure nodes of the basis structure contain further substructures holding the data elements. The data elements represent the lowest level of the hierarchical data structure and are here referred to as *atomic elements*. These contain the data and metadata of all laboratory experiments.

#### Basic structure

The basic structure represents the backbone of the hierarchical data structure and ties the substructures together. The substructure dataset.tst introduces an additional structure level which holds the substructures containing the laboratory test data. The tree view below briefly summarises the basic data structure’s elements as used for the datasets of Test series 1.



#### Substructures

The substructures (e.g., dataset.meta_set, dataset.aut, dataset.s01) contain thematically related data elements, the *atomic elements*. The substructures are present in the data set either as a scalar substructure (cardinality *C* = 1, e.g., dataset.meta_set) or as an indexed substructure sequence (cardinality *C* ≥1, e.g., dataset.dev). In substructure sequences, the composition of the data elements is identical. These only differ in the data stored in the fields of the respective data elements.

A common feature of all substructures and data elements are the fields *object* (e.g., dataset.spm.obj) and *version* (e.g., dataset.spm.ver). The *object* field contains a unique identifier representing the format of the substructure. The *version* field contains a tuple of two unsigned integers representing the version number of the structure format. By these two data fields, it is possible to make future adaptations and corrections visible and to ensure compatibility with analysis algorithms relying on a specific structure format.

#### Atomic elements

The atomic elements represent the lowest structural level. They are made up of several data fields and can, therefore, be regarded as complex data types. The composition of the data fields of these elements depends on their use. The three structure types defined below are used in the data sets of this study.

• Atomic data element (ADE): This element is mainly used to store numerical data (e.g., measurement data) and contains the fields *tag* (ADE.t), *value type* (ADE.vt), *value* (ADE.v), *physical unit* (ADE.u) and *description* (ADE.d). The field ADE.vt contains a data type descriptor and enables the correct interpretation of the value stored in the field ADE.v. The field ADE.u provides information about the physical unit of the value stored in ADE.v.

• Atomic attribute element (AAE): This element is used exclusively for storing metadata in the form of strings (character arrays) and contains the fields *tag* (AAE.t), *value* (AAE.v) and *description* (AAE.d). The AAE.v field can consist of either a single string or a sequence of strings (GNU Octave cell array).

• Atomic reference element (ARE): This element is used exclusively for referencing substructures and contains the fields *tag* (ARE.t), *identifier* (ARE.i), *reference* (ARE.r) and *description* (ARE.d). This element prevents the need to create copies of substructures used multiple times. For example, a specimen that is used in several consecutive tests. The field ARE.r contains a sequence of character strings that map the structure path to the referenced substructure (e.g., *dataset.spm*). The field ARE.i contains the unique numerical identifier of the referenced substructure. All referable substructures contain an ADE with the object name d01, whose field *value* (d01.v) contains the unique numerical identifier.

The fields *tag* and *description* are present in all three variants and contain the data element’s short name and the content’s description, respectively. A detailed illustration of the datasets’ structure elements of Test series 1, please find in Supplementary Fig. 1. The datasets’ structure of the reference tests of Test series 5, Test series 6 and Test series 7, please find in Supplementary Fig. 2. Both figures can be found in the ‘Supplementary Information Document’ linked to this paper. Application examples in section ‘Usage Notes’ explain how to access the data in that structures.

## Technical Validation

This section is dedicated to validating the technical quality of the ultrasound measuring results. The validation aims to show that the signal data reproducibly reflects the underlying physical laws of sound propagation. For this purpose, the ultrasound measurement data from the datasets of Test series 5, Test series 6 and Test series 7 are used, since the properties of the materials used are known. The selected datasets and the analysis methods are detailed in subsection ‘Analysis methods for the technical validation’.

During the validation procedure, two quantitative metrics are used to justify whether the signal data reflects the fundamental physical laws of sound propagation — the ensemble average of signal sequences and the deviation from the ensemble average. The ensemble average is a robust measure to quantify the temporal localisation of the first incoming sound wave and its reflections as well as the shape of the signal curve. The distribution of the deviations from the ensemble average provides information on the reproducibility of the ultrasound tests. A low distribution spread indicates low signal response variability and is therefore a reasonable repeatability measure.

Due to measurement tolerances and procedural errors, the analysis results are not expected to accurately reproduce the literature-based predictions for the sound travel time also referred to as time-of-flight. However, those predictions should be within the estimated tolerance limits. The following effects and behavioural patterns should be evident in the analysis results.

1. The sound travel time observed is within the tolerance range of literature-based estimates considering procedural and measuring errors.

2. The relation between the sound travel time and the ultrasonic measuring distance is linear.

3. The sound travel times of the reflections of the sound wave are odd integer multiples of the sound travel time of the first appearance of the sound wave.

4. For all materials, the signal amplitudes decrease with increasing ultrasonic measuring distance.

5. For air (an approximately ideal gas), the maximum amplitude of the first incoming sound wave and the maximum amplitudes of the reflections of this sound wave are decreasing linearly.

6. Using the same test parameters, repeated test execution leads to similar results.

The graphical representations of the analysis results are summarised in the following list, sorted by test series and ultrasonic measuring distance.

**Fig. 7:**
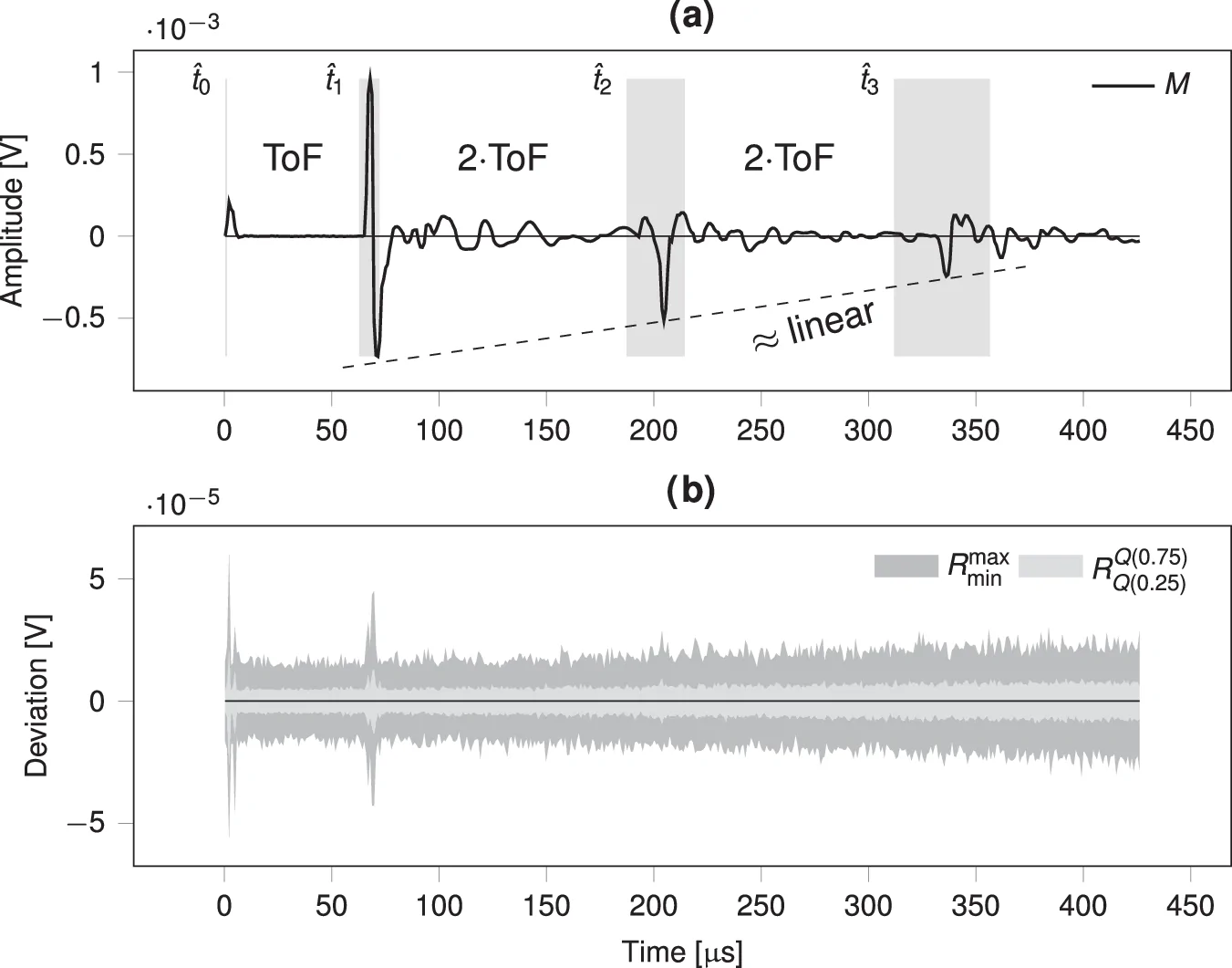
Test series 5, reference tests on air – primary wave, *D* = 25 mm. **(7a)** Ensemble average *M*. **(7b)** Deviation from ensemble average *R*.

**Fig. 8:**
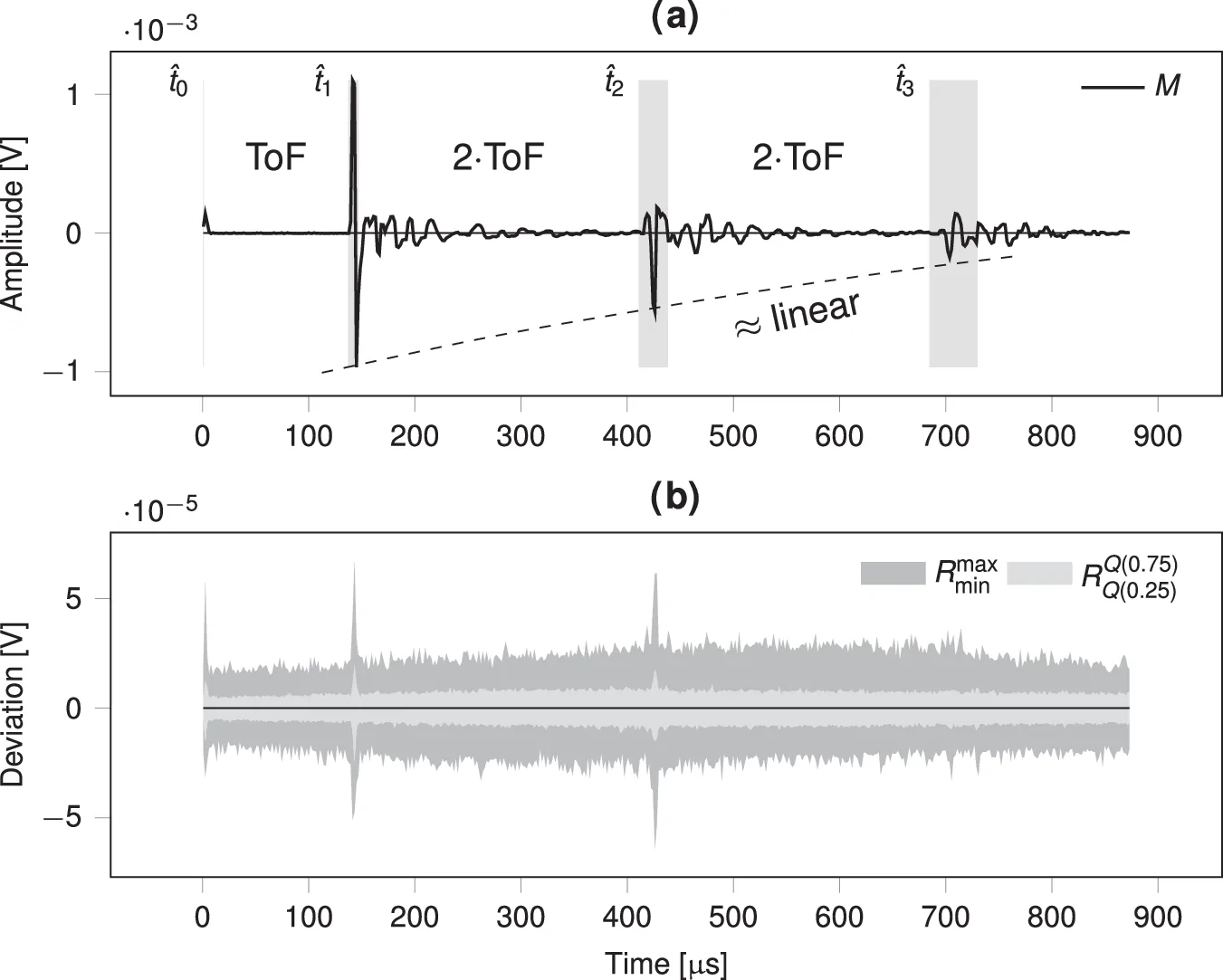
Test series 5, reference tests on air – primary wave, *D* = 50 mm. **(8a)** Ensemble average *M*. **(8b)** Deviation from ensemble average *R*.

**Fig. 9:**
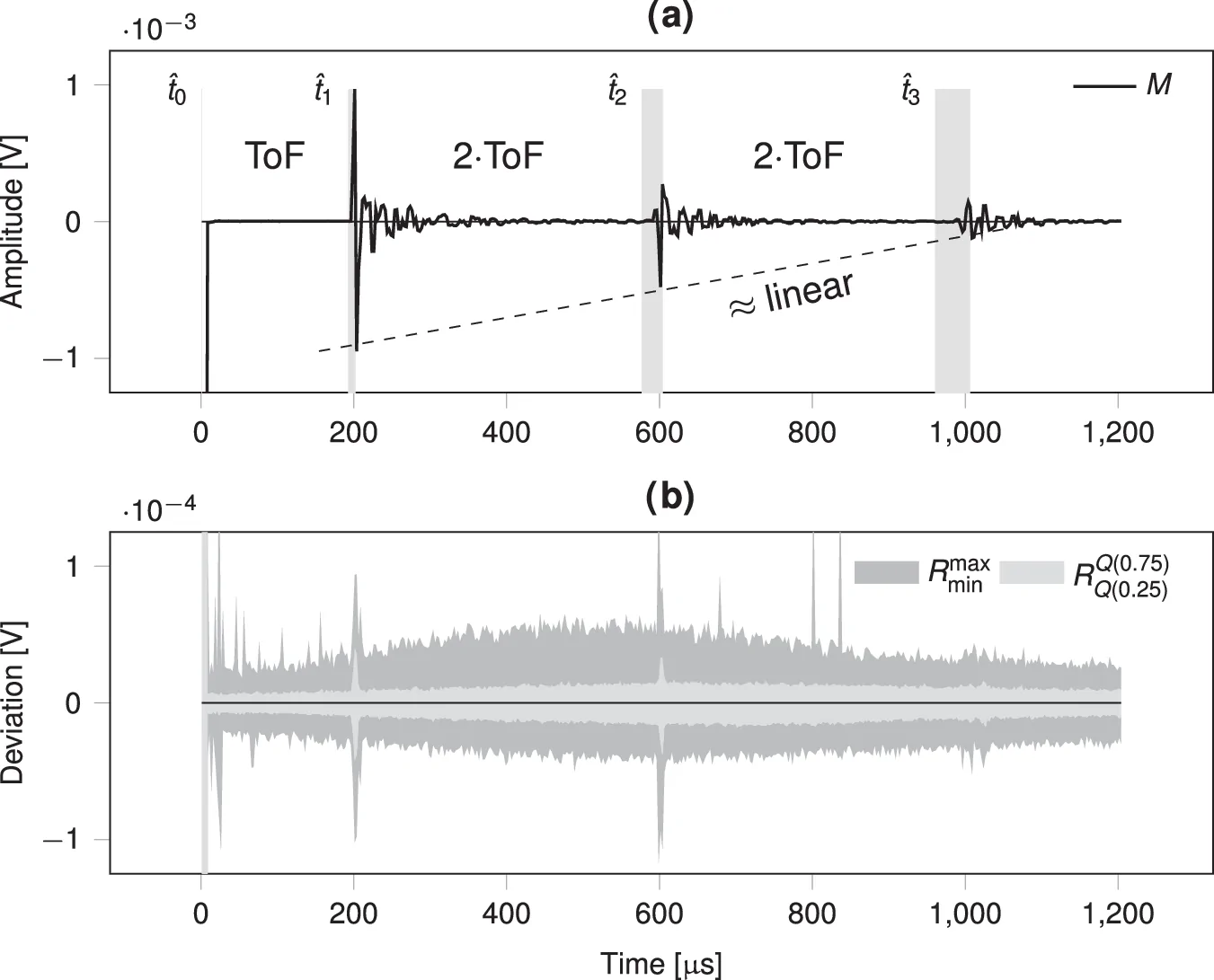
Test series 5, reference tests on air – primary wave, *D* = 50 mm. **(9a)** Ensemble average *M*. **(9b)** Deviation from ensemble average *R*.

**Fig. 10:**
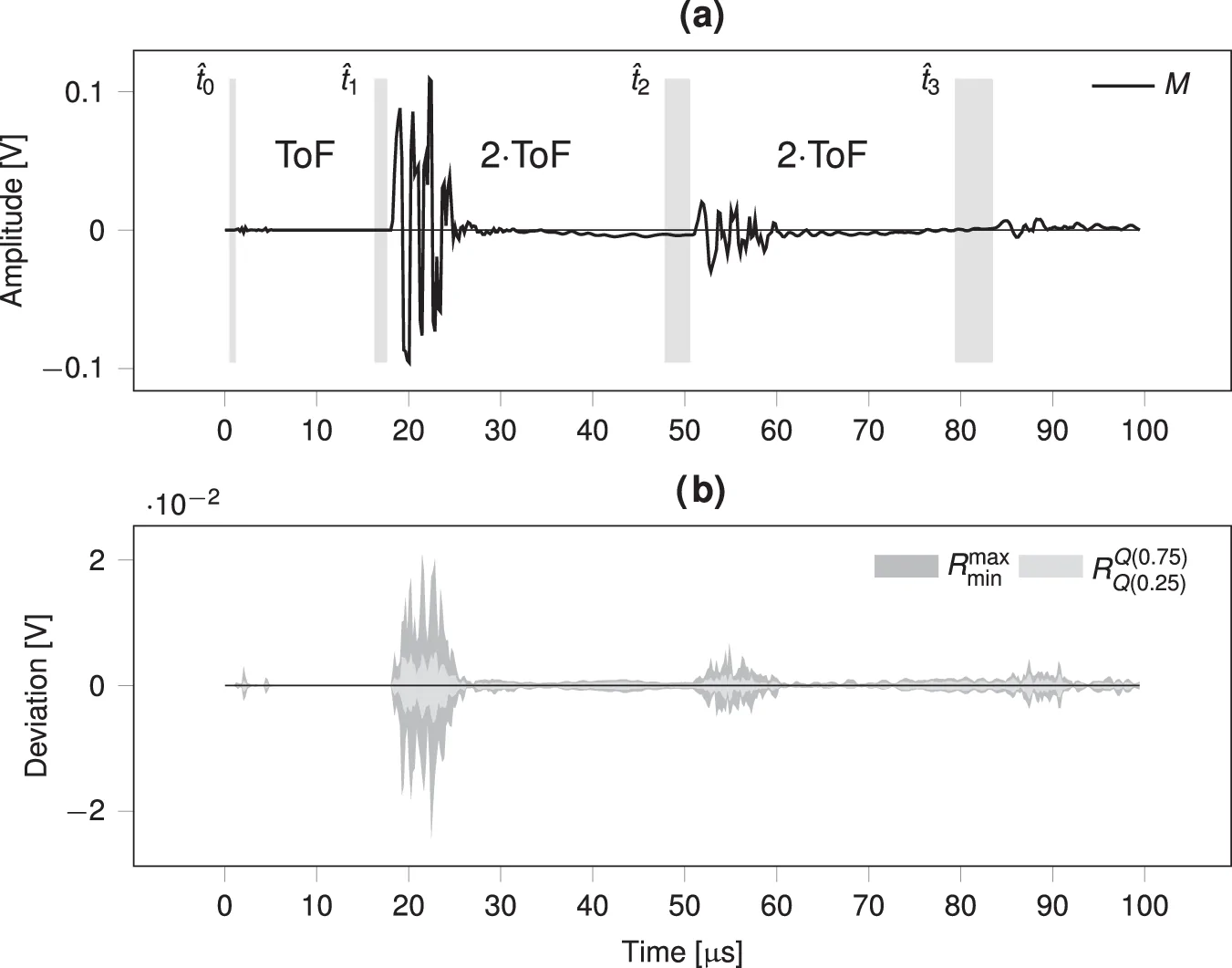
Test series 6, reference tests on water – primary wave, *D* = 25 mm. **(10a)** Ensemble average *M*. **(10b)** Deviation from ensemble average *R*.

**Fig. 11:**
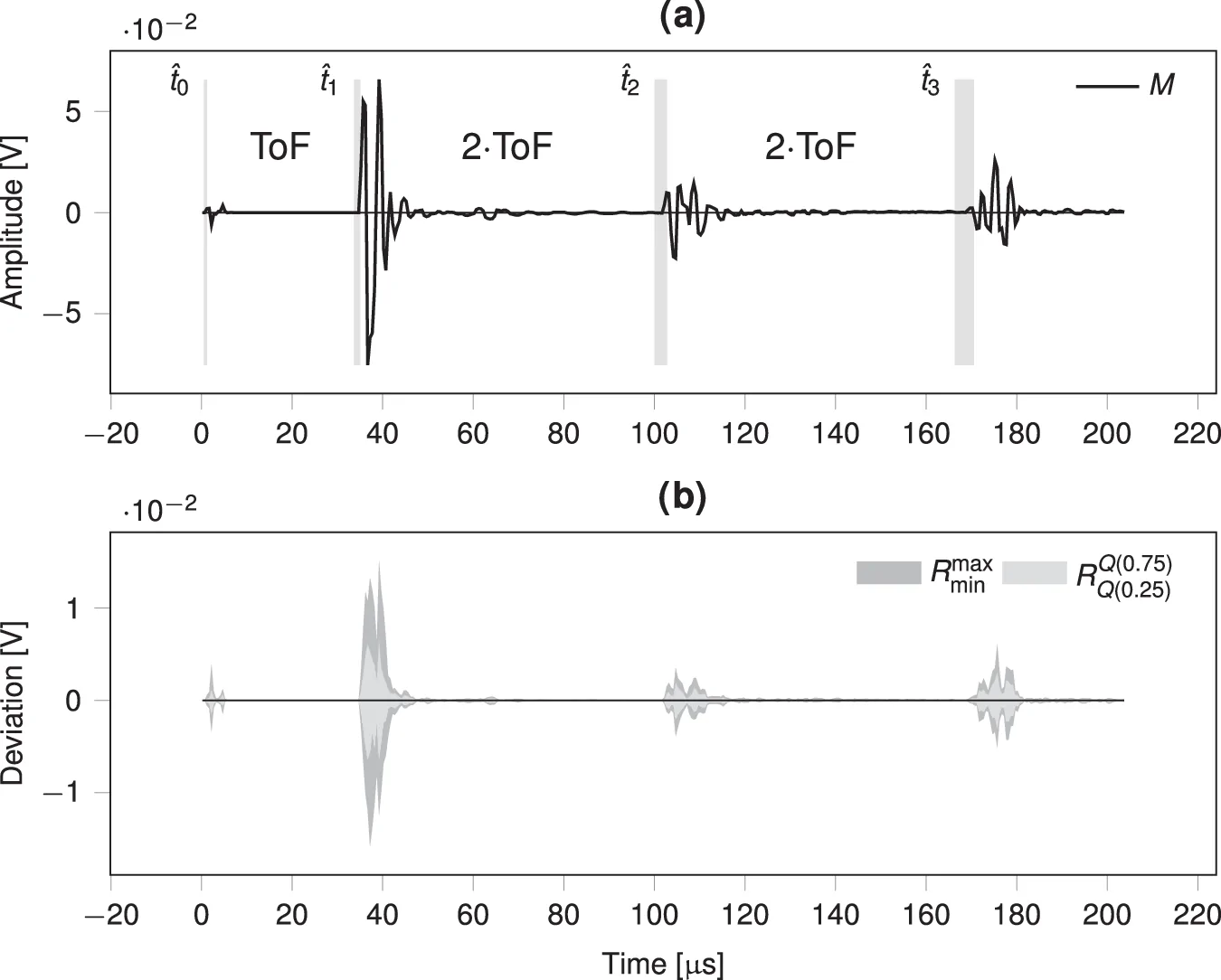
Test series 6, reference tests on water – primary wave, *D* = 50 mm. **(11a)** Ensemble average *M*. **(11b)** Deviation from ensemble average *R*.

**Fig. 12:**
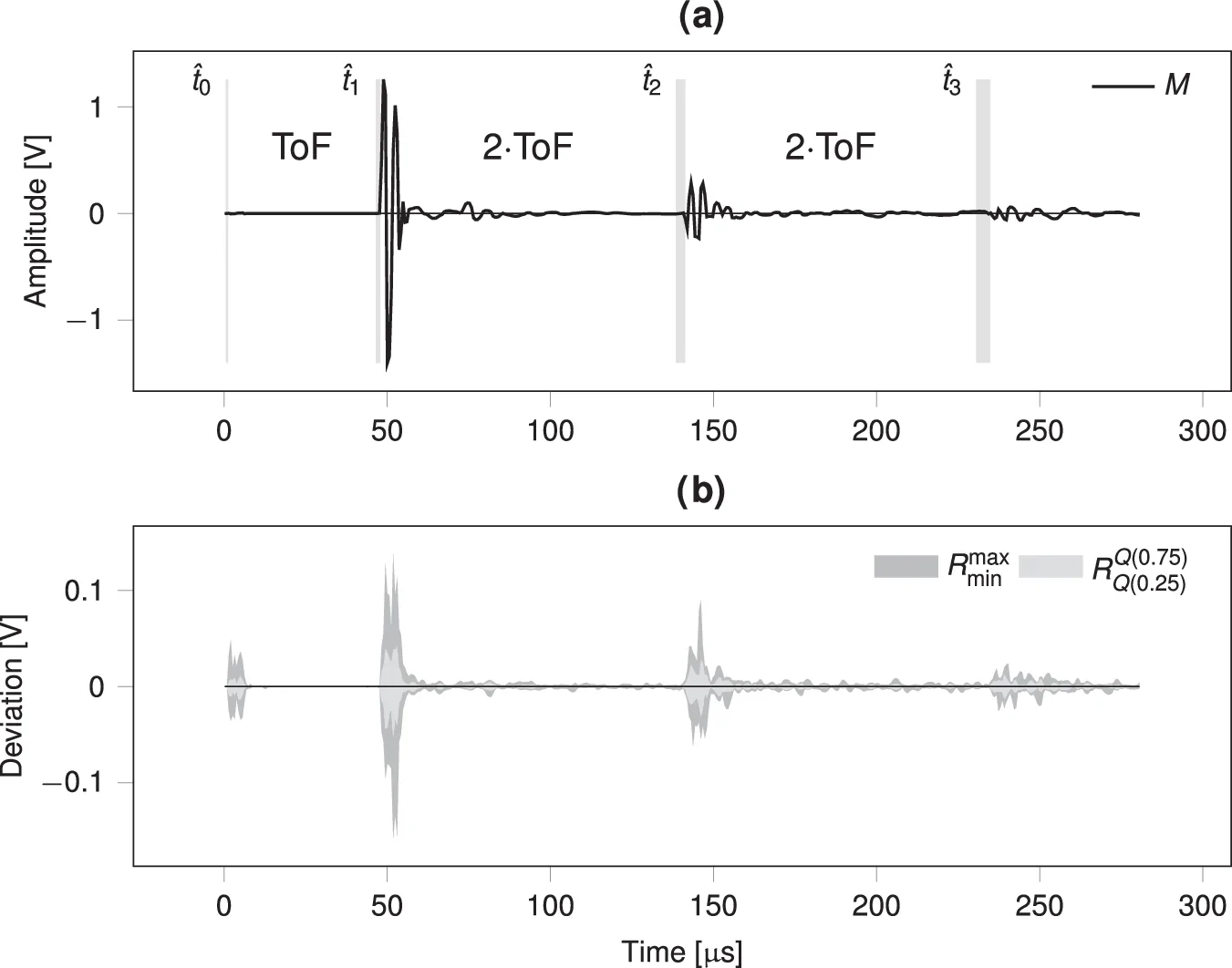
Test series 6, reference tests on water – primary wave, *D* = 70 mm. **(12a)** Ensemble average *M*. **(12b)** Deviation from ensemble average *R*.

**Fig. 13:**
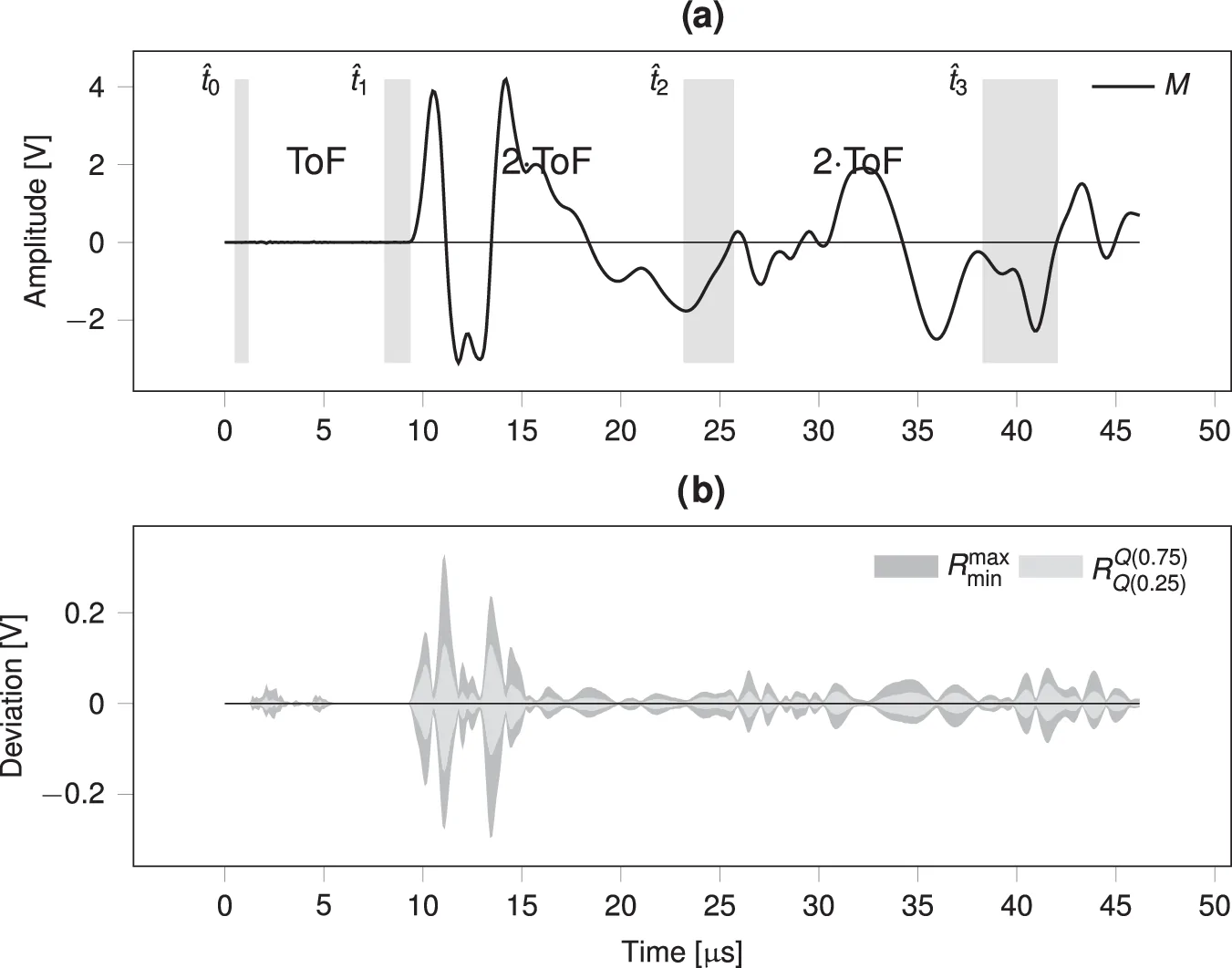
Test series 7, reference tests on aluminium cylinder – primary wave, *D* = 50 mm. **(13a)** Ensemble average *M*. **(13b)** Deviation from ensemble average *R*.

**Fig. 14:**
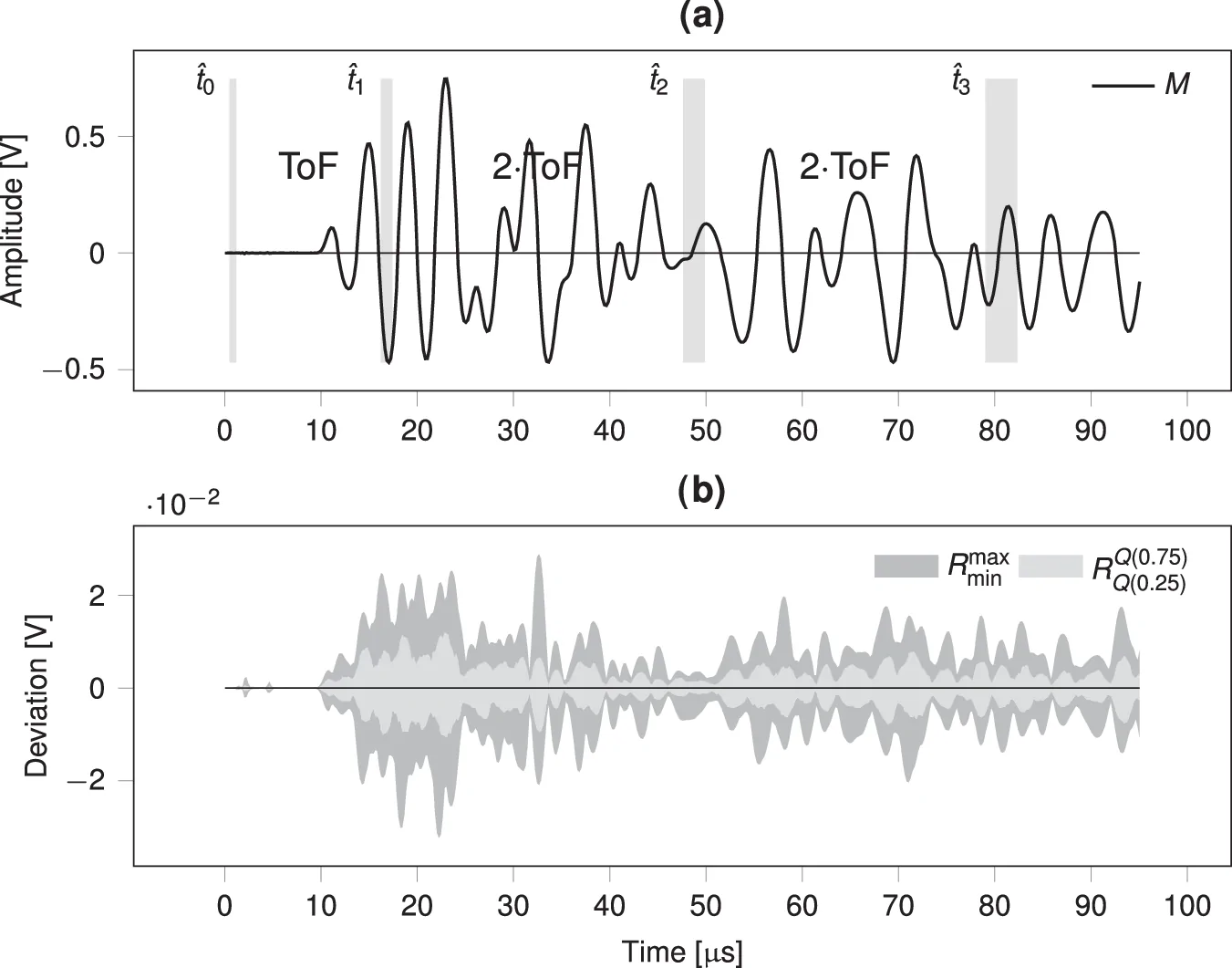
Test series 7, reference tests on aluminium cylinder – secondary wave, *D* = 50 mm. **(14a)** Ensemble average *M*. **(14b)** Deviation from ensemble average *R*.

• Test series 5, reference tests on air, primary wave’ signal response

— *D* = 25 mm: Fig. 7

— *D* = 50 mm: Fig. 8

— *D* = 70 mm: Fig. 9

• Test series 6, reference tests on water, primary wave’ signal response

— *D* = 25 mm: Fig. 10

— *D* = 50 mm: Fig. 11

— *D* = 70 mm: Fig. 12

• Test series 7, reference tests on aluminium cylinder, *D* = 50 mm

— Primary wave’ signal response: Fig. 13

— Secondary wave’ signal response: Fig. 14

The illustrations of the analysis results show that the predicted sound travel times can be read from the ensemble average *M* of the ultrasound signal sequences. They also show that almost all behavioural patterns listed above are met, with the exception of criterion No. 4.

According to criterion No. 4, a reciprocal relation between ultrasonic measuring distance *D* and the maximum signal amplitude of the first appearance of the sound wave is expected. The analysis results of the reference tests on air and water do not satisfy this expectation. For the reference tests on water, the maximum amplitude (${M}_{\max }\approx 1$ V) shown in Fig. 12 (*D* = 70 mm) is larger than the maximum amplitude (${M}_{\max }\approx 0.07$ V) shown in Fig. 8 (*D* = 50 mm). Furthermore, for the reference tests on air, the maximum amplitude of *M* seems to be independent of the ultrasonic measuring distance of *D* because the maximum amplitudes of (${M}_{\max }\approx 0.001$ V) in Fig. 7 (*D* = 25 mm), Fig. 8 (*D* = 50 mm) and Fig. 9 (*D* = 70 mm) are almost equal. This contradictory behaviour is due to the automatic amplitude pre-amplification of the oscilloscope mentioned in subsection ‘Data acquisition’. Nevertheless, this criterion is met within the ensemble average of a signal sequence, since the maximum amplitudes of the reflections of the sound wave become smaller with increasing distance.

The analysis results for the aluminium cylinder are admittedly difficult to read. Particularly apparent becomes the fact mentioned in subsection ‘Experimental setup’ that the signal response of the secondary wave (shear wave) in Fig. 14 is superimposed by the signal response of the primary wave (compression wave). Conversely, the same applies to the signal response of the primary wave shown in Fig. 13. However, the sound travel time of the primary wave and the deviation from the ensemble average are readable.

The reproducibility of the ultrasound tests appears to be consistent. The minimum and maximum deviations are in the range of one-twentieth of the maximum amplitude of the ensemble average in all cases shown. Whether that is sufficient for a particular application can only be justified within the context of that application.

## Usage Notes

This section describes how to access the data records’ content and to use the included binary datasets. The following explains how to extract the datasets from the data records’ compressed TAR archives, access the data structure in the datasets, and export and visualise signal data. The end of this section provides instructions to reproduce the analysis results illustrated in section ‘Technical Validation’. The descriptions below refer to the software provided by the author of this study (see ‘Code Availability’) but also to freely available open-source software.

### Extracting datasets from compressed TAR archives

After downloading the data records from the repository^11^, the datasets are available in compressed TAR archives. To access the contained datasets, extract them from the archives first. Under Linux, the command line tool GNU tar is the simplest option. The following command decompresses and extracts the datasets from the TAR archives into the folder ‘ts1_dataset’ using the GNU bash^34^ command line interface.


$ tar -xzf ts1_dataset.tar.xz


### Accessing the data in a data set

Since the datasets were written in the binary file format of GNU Octave, accessing them with this program is the most convenient option. Access to the structure elements’ data is possible using the following commands on the GNU Octave command line interface. Please note that lines beginning with a # sign are comments which refer to the subsequent line.

> > #Load dataset and assign entire structure to variable ds

> > ds = load('/path/to/dataset/file.oct', 'dataset').dataset;

> > #Assign the data of signal 12 from channel 1 to array s

> > s = ds.tst.s06.d13.v(:,12);

> > # Assign measurement unit of the signal data to variable u

> > u = ds.tst.s06.d13.u;

Please see section ‘Data Records’ for an elaborate description of all structure elements. A general introduction^35^ on using structures in GNU Octave is also available online.

### Extracting data from a data set

The access to the datasets’ measurement data and metadata is not limited to GNU Octave. To use the data stored in the datasets in other programs, they must be converted to other common file formats first. For this purpose, the script collection ‘Data set exporter 1.1’^36^ is provided which includes an example script as well (see file dsexporter_examples.m). That script can be used to copy-paste code or as a basis to write application-specific data export scripts.

The following examples show how to initialise that program, export ultrasound signal data and specimen temperature data as a comma-separated value file (CSV), and to convert the metadata into the JSON structure format or the LaTeX format. Please note, that the function ‘init()’ must be called once before executing the other commands.

> > # (1) Initialise program 'Dataset Exporter'

> > init();

> > # (2) Load export settings into variable ES

> > ES = dsexporter_settings();

> > # (3) Define the output directory path ODP

> > ODP = '/path/to/output/directory';

> > # (4) Define the data set file path IFP

> > IFP = '/path/to/dataset/dataset.oct';

> > # (5) Export signal 10 from channel 1 to CSV file

> > dsexporter_sig2csv(ES, ODP, IFP, 1, 10);

> > # (6) Export specimen temperature data to a CSV file

> > dsexporter_tem2csv(ES, ODP, IFP, 1);

> > # (7) Export data set metadata to a JSON file

> > dsexporter_substruct2json(ES, ODP, IFP, 'meta_set');

> > # (8) Export data set metadata to a LaTeX file

> > dsexporter_substruct2tex(ES, ODP, IFP, 'meta_set');

### Visualising data from a data set

Data visualisation is one of the most important tasks in signal analysis. To simplify that task, the script collection Data set viewer 1.0^37^ is provided. The following examples show how to initialise the program, plot signal and temperature data, and render a sequence of ultrasound signals into an MP4 video file.

> > # (1) initialise program 'Dataset Viewer'

> > init();

> > # (2) load display settings

> > VS = dsviewer_settings();

> > # (3) define data set input file path

> > IFP = '/path/to/dataset/dataset.oct';

> > # (4) define temporary directory to create MP4 videos

> > TMP = '/path/to/temp';

> > # (4) display signal 100 from channel 1, 2D plot

> > dsviewer_2d(IFP, VS, 1, 100);

> > # (5) plot all signals from channel 1, 3D plot

> > dsviewer_3d(IFP, VS, 1);

> > # (6) render MP4 video of all signals in data set

> > dsviewer_mp4(IFP, TMP, VS);

> > # (7) display temperature data from channel 1

> > dsviewer_tem(IFP, VS, 1);

### Converting datasets to other binary file formats

Other widely used binary file formats to store structured data are the MatLAB^38^ binary file format and the binary, hierarchical data format HDF5^39^. The following examples show how to perform that conversion using the GNU Octave command line interface. Furthermore, the script collection Data set exporter 1.1^36^ includes two GNU Octave scripts (‘dsexporter_convert_to_matlab.m’, ‘dsexporter_convert_to_hdf5.m’) that allow for easy conversion of the data sets.

> > # load dataset (GNU Octave's binary format)

> > ds = load('/path/to/dataset.oct', 'dataset').dataset;

> > # convert dataset to the MatLAB binary file format, version 6

> > save('-v6', 'dataset_matlabv6.mat', 'ds');

> > # convert dataset to the MatLAB binary file format, version 7

> > save('-v7', 'dataset_matlabv7.mat', 'ds');

> > # convert dataset to the binary hierarchical data format HDF5

> > save('-hdf5', 'dataset_hdf.hdf5', 'ds');

> > # convert to MatLAB format using a script

> > dsexporter_convert_to_matlab(... '/path/to/input/dataset.oct', '/path/to/output/dataset.mat');

> > # convert to HDF5 format using a script

> > dsexporter_convert_to_hdf5(... '/path/to/input/dataset.oct', '/path/to/output/dataset.hdf5');

### Reproducing the analysis results

The analysis results shown in section ‘Technical Validation’ were compiled using GNU Octave command scripts. First, download the TAR archives of Test series 5^21^, Test series 6^22^ and Test series 7^23^ from the repository and extract the datasets from that archives as described above.

Second, download the command script archive^40^ from the repository and extract the files from the archive into a working directory. See also section ‘Code Availability’.

Third, adjust the below-described variables in function file anadd_param.m such that they point to the location of the previously extracted datasets (.oct).


# full path, parent dirctory of the subdirectories# 'ts5_datasets', 'ts6_datasets', 'ts7_datasets'r_ds.paths.dssrc = /home/acme/science/data;# test series 5 *.oct files, subdirectory namer_ds.paths.ts5oct = 'ts5_datasets';# test series 6 *.oct files, subdirectory namer_ds.paths.ts5oct = 'ts6_datasets';# test series 7 *.oct files, subdirectory namer_ds.paths.ts5oct = 'ts7_datasets';


Finally, the commands listed below allow to reproduce the analysis results from the reference tests’ datasets using the GNU Octave command line interface.

> > # initialise program

> > anadd_init();

> > # compile results and export them to LaTeX text files

> > anadd_analyse();

## Supplementary information

Supplementary Information Document concerning Experimental study on cement paste using the ultrasonic pulse transmission method

## Data Availability

The data records that conclude the datasets detailed in section ‘Data Records’ are made available at *TU Graz Repository*. To ensure sustained use, the data records and the datasets are licensed under the Creative Commons Attribution 4.0 International (CC BY 4.0) license^10^. • **Location**.^11^ TU Graz Repository, https://doi.org/10.17616/R31NJMYL, https://repository.tugraz.at/ • **Data record 1**.^30^ Test series 1, https://doi.org/10.3217/bhs4g-m3z76, • **Data record 2**.^21^ Test series 5, https://doi.org/10.3217/bjkrj-pg829, • **Data record 3**.^22^ Test series 6, https://doi.org/10.3217/hn7we-q7z09, • **Data record 4**.^23^ Test series 7, https://doi.org/10.3217/w3mb5-1wx17,

## Code availability

All datasets referenced in this work were compiled from the raw data into the binary file format of GNU Octave using command scripts. Therefore, compiling the datasets is not necessary. Nevertheless, those scripts help to comprehend the details of the compilation process.

Further script collections are provided that simplify access to the binary datasets and make it easier to visualise the signal data therein. In addition, the script collection used to compile and reproduce the analysis results presented in section ‘Technical Validation’ is supplied.

All script collections listed below were implemented and tested with GNU Octave on the Debian 11^41^ (Linux) operating system. For extensibility and sustainable use, the program code provided is licensed under the MIT^42^ license and made available at the TU Graz repository^11^. Usage examples for that script collections, please find in section ‘Usage Notes’.

• Data set compiler 1.2^25^ is a tool for compiling the raw data into binary datasets with a hierarchical data structure. In addition, this tool also contains the metadata databases used in the data set compilation process.

• Data set exporter 1.1^36^ is a tool for exporting data from the binary datasets. The following file formats are supported: (1) comma-separated text format, (2) the JSON structure format, and (3) the LaTeX text format, (4) and audio files in the waveform audio file format (.wav).

• Data set viewer 1.0^37^ is a tool for visualising data from the binary datasets. The following functions are supported: (1) 2D plots of ultrasonic signal data; (2) 2D plots of the temperature data of the specimens; (3) 3D plots of ultrasound signal sequences and (4) rendering ultrasound signal sequences to the MP4 format.

• The script collection^40^ used to compile the analysis results for the technical validation.

• GNU Octave^33^: scientific programming language.

• GNU tar^31^: command line interface (CLI) tool to handle TAR archives.

• Gzip^32^: CLI tool to handle ZIP archives.

• ffmpeg^43^: CLI tool to create and manipulate MPEG video files.

• shasum^44^: CLI tool to print or check SHA checksums. This tool is only needed to compile the binary datasets.

This open-source software is available for Linux and other operating systems (e.g., Microsoft Windows or MacOS). For Microsoft Windows users, using the freely available software 7-zip^45^ is suggested instead of GNU tar and Gzip.
