# Supplementary material for: Experimental study on cement paste using the ultrasonic pulse transmission method
Source: Sci Data. 2026 Jul 10;13:1502. doi: 10.1038/s41597-026-07659-5 (PMC13728127; doi:10.1038/s41597-026-07659-5)
Supplement: Supplementary file 1 [file 41597_2026_7659_MOESM1_ESM.pdf]

Supplementary Information Document

concerning

Experimental study on cement paste using the ultrasonic pulse transmission method

Jakob Harden*

May 10,2026

Table of Contents

Supplementary Figure 1 2
Supplementary Figure 2 3

*Graz University of Technology, Faculty of Civil Engineering Sciences, Institute of Technology and Testing of Construction Materials, Graz, Austria. email: jakob.harden@tugraz.at

(a)

- dataset … structure root, $C = 1$
  - **Test series metadata**
  - meta_ser … test series metadata, $C = 1$
    - obj … object type descriptor, 'struct_meta_ser', string, $C = 1$
    - ver … version number [major, minor], unsigned 16bit-integer tuple, $C = 1$
    - r01 … author reference $\mapsto$ dataset.aut, ARE, $C = 1$
    - r02 … license reference $\mapsto$ dataset.lic, ARE, $C = 1$
    - d01 … series identification number, unique ID, ADE, $C = 1$
    - a01 … series designation, short text or code, AAE, $C = 1$
    - a02 … series name, AAE, $C = 1$
    - a03 … series description, AAE, $C = 1$
    - a04 … abstract, e.g. introductory text, AAE, $C = 1$
    - a05 … context, e.g. PhD thesis, AAE, $C = 1$
    - a06 … start date, e.g. date of first test, AAE, $C = 1$
    - a07 … end date, e.g. date of last test, AAE, $C = 1$
  - **Data set metadata**
  - meta_set … data set metadata, $C = 1$
    - obj … object type descriptor, 'struct_meta_set', string, $C = 1$
    - ver … version number [major, minor], unsigned 16bit-integer tuple, $C = 1$
    - r01 … author reference $\mapsto$ dataset.aut, ARE, $C = 1$
    - r02 … test series reference $\mapsto$ dataset.meta_ser, ARE, $C = 1$
    - r03 … geo-location reference $\mapsto$ dataset.loc, ARE, $C = 1$
    - r04 … license reference $\mapsto$ dataset.lic, ARE, $C = 1$
    - d01 … data set identification number, unique ID, ADE, $C = 1$
    - a01 … data set designation, short text or code, AAE, $C = 1$
    - a02 … data set name, AAE, $C = 1$
    - a03 … description, general, AAE, $C = 1$
    - a04 … description, abstract, AAE, $C = 1$
    - a05 … description, methods, AAE, $C = 1$
    - a06 … description, table of contents, AAE, $C = 1$
    - a07 … data set creator name, AAE, $C = 1$
    - a08 … data set collector name, AAE, $C = 1$
    - a09 … data set copyrighter name, AAE, $C = 1$
    - a10 … date created, AAE, $C = 1$
    - a11 … date collected, AAE, $C = 1$
    - a12 … date copyrighted, AAE, $C = 1$
    - a13 … data set size, number of files, AAE, $C = 1$
    - a14 … data set file format, AAE, $C = 1$
    - a15 … data set version, AAE, $C = 1$
    - a16 … data set context, AAE, $C = 1$
    - a17 … data set rawdata directory, AAE, $C = 1$
    - a18 … data set rawdata archive, AAE, $C = 1$
  - **Location information**
  - loc … geo-location information, $C \geq 1$
    - obj … object type descriptor, 'struct_loc', string, $C = 1$
    - ver … version number [major, minor], unsigned 16bit-integer tuple, $C = 1$
    - d01 … location identification number, unique ID, ADE, $C = 1$
    - d02 … geolocation, latitude and longitude, ADE, $C = 1$
    - a01 … country, AAE, $C = 1$
    - a02 … state or province, AAE, $C = 1$
    - a03 … city, AAE, $C = 1$
    - a04 … zip code, postal code, AAE, $C = 1$
    - a05 … street name, AAE, $C = 1$
    - a06 … house number, AAE, $C = 1$
    - a07 … location description, AAE, $C = 1$
  - **License information**
  - lic … license information, $C \geq 1$
    - obj … object type descriptor, 'struct_lic', string, $C = 1$
    - ver … version number [major, minor], unsigned 16bit-integer tuple, $C = 1$
    - r01 … author reference $\mapsto$ dataset.aut, ARE, $C = 1$
    - d01 … license identification number, unique ID, ADE, $C = 1$
    - a01 … license designation, short text or code, AAE, $C = 1$
    - a02 … rights holder, AAE, $C = 1$
    - a03 … rights description, AAE, $C = 1$
    - a04 … rights URI, AAE, $C = 1$
    - a05 … rights identifier scheme, AAE, $C = 1$
    - a06 … rights identifier scheme URI, AAE, $C = 1$
    - a07 … license description, AAE, $C = 1$
    - a08 … license icon, AAE, $C = 1$
    - a09 … license id, AAE, $C = 1$
  - **Author information**
  - aut … author information, $C \geq 1$
    - obj … object type descriptor, 'struct_aut', string, $C = 1$
    - ver … version number [major, minor], unsigned 16bit-integer tuple, $C = 1$
    - d01 … author identification number, unique ID, ADE, $C = 1$
    - a01 … full name, AAE, $C = 1$
    - a02 … given name, first name, AAE, $C = 1$
    - a03 … family name, surname, AAE, $C = 1$
    - a04 … initials, AAE, $C = 1$
    - a05 … title before the name (prefix), AAE, $C = 1$
    - a06 … title behind the name (suffix), AAE, $C = 1$
    - a07 … organization name, AAE, $C = 1$
    - a08 … department name, AAE, $C = 1$
    - a09 … role in organization/department, AAE, $C = 1$
    - a10 … country, AAE, $C = 1$
    - a11 … state or province, AAE, $C = 1$
    - a12 … city, AAE, $C = 1$
    - a13 … zip code, postal code, AAE, $C = 1$
    - a14 … street name, AAE, $C = 1$
    - a15 … email address, AAE
    - a16 … name identifier type, AAE, $C = 1$
    - a17 … name identifier type uri, AAE, $C = 1$
    - a18 … name identifier, AAE, $C = 1$
    - a19 … author description, AAE, $C = 1$
  - **Testing device information**
  - dev … testing device information, structure array, $C \geq 1$
    - obj … object type descriptor, 'struct_dev', string, $C = 1$
    - ver … version number [major, minor], unsigned 16bit-integer tuple, $C = 1$
    - d01 … device identification number, unique ID, ADE, $C = 1$
    - a01 … device name, AAE, $C = 1$
    - a02 … vendor name, AAE, $C = 1$
    - a03 … product name, AAE, $C = 1$
    - a04 … device category, AAE, $C = 1$
    - a05 … device usage, AAE, $C = 1$
    - a06 … device description, AAE, $C = 1$
    - s01 … device properties (optional), array of AAE, $C \geq 0$
  - **Material information**
  - mat … test material information, structure array, $C \geq 1$
    - obj … object type descriptor, 'struct_mat', string, $C = 1$
    - ver … version number [major, minor], unsigned 16bit-integer tuple, $C = 1$
    - d01 … material identification number, unique ID, ADE, $C = 1$
    - a01 … material name, AAE, $C = 1$
    - a02 … vendor name, AAE, $C = 1$
    - a03 … product name, AAE, $C = 1$
    - a04 … material category, AAE, $C = 1$
    - a05 … material description, AAE, $C = 1$
    - a06 … material storage place, AAE, $C = 1$
    - a07 … material storage condition, AAE, $C = 1$
    - s01 … material properties (optional), array of AAE, $C = 1$
  - **Mixture recipe**
  - rec … mixture/blend recipe definition, $C = 1$
    - obj … object type descriptor, 'struct_rec', string, $C = 1$
    - ver … version number [major, minor], unsigned 16bit-integer tuple, $C = 1$
    - d01 … recipe identification number, unique ID, ADE, $C = 1$
    - a01 … recipe designation, short text or code, AAE, $C = 1$
    - s01 … mixture/blend components, structure array, $C \geq 1$
      - obj … object type descriptor, 'struct_mix_component', string, $C = 1$
      - ver … version number [major, minor], unsigned 16bit-integer tuple, $C = 1$
      - r01 … material reference $\mapsto$ dataset.mat, ARE, $C = 1$
      - d01 … material weight, ADE, $C = 1$
      - a01 … description, AAE, $C = 1$
    - s02 … mixture parameters, water-cement ratio, array of ADE, $C = 1$
  - **Mixing procedure**
  - mix … mixing procedure, $C = 1$
    - obj … object type descriptor, 'struct_mix', string, $C = 1$
    - ver … version number [major, minor], unsigned 16bit-integer tuple, $C = 1$
    - r01 … author reference $\mapsto$ dataset.aut, ARE, $C = 1$
    - r02 … device reference $\mapsto$ dataset.dev, ARE, $C = 1$
    - r03 … recipe reference $\mapsto$ dataset.rec, ARE, $C = 1$
    - r04 … geo-location reference $\mapsto$ dataset.loc, ARE, $C = 1$
    - d01 … mixture identification number, unique ID, ADE, $C = 1$
    - d02 … date and time, seconds since epoch (UTC), ADE, $C = 1$
    - d03 … mixing time, ADE, $C = 1$
    - d04 … mixer speed level, ADE, $C = 1$
    - d05 … mixer speed level, ADE, $C = 1$
    - d06 … mixer attachment speed, ADE, $C = 1$
    - a01 … operator name, AAE, $C = 1$
    - a02 … procedure description, AAE, $C = 1$
    - a03 … general description, AAE, $C = 1$
  - **Specimen information**
  - spm … specimen information, specimen I and II, structure array, $C \geq 1$
    - obj … object type descriptor, 'struct_spm_paste', string, $C = 1$
    - ver … version number [major, minor], unsigned 16bit-integer tuple, $C = 1$
    - r01 … author reference $\mapsto$ dataset.aut, ARE, $C = 1$
    - r02 … mixture reference $\mapsto$ dataset.mix, ARE, $C = 1$
    - r03 … device reference $\mapsto$ dataset.dev, ARE, $C = 1$
    - r04 … geo-location reference $\mapsto$ dataset.loc, ARE, $C = 1$
    - d01 … specimen identification number, unique ID, ADE, $C = 1$
    - d02 … date and time, seconds since epoch (UTC), ADE, $C = 1$
    - a01 … specimen designation, short text or code, AAE, $C = 1$
    - a02 … operator name, AAE, $C = 1$
    - a03 … procedure description, AAE, $C = 1$
    - a04 … general description, AAE, $C = 1$
  - tst … test collection, $C = 1$

(b)

- tst … test collection, $C = 1$
  - obj … object type descriptor, 'struct_test', string, $C = 1$
  - ver … version number [major, minor], unsigned 16bit-integer tuple, $C = 1$
  - **Fresh paste density**
  - s01 … fresh paste density, $C \geq 0$
    - obj … object type descriptor, 'struct_test_fpd', string, $C = 1$
    - ver … version number [major, minor], unsigned 16bit-integer tuple, $C = 1$
    - r01 … author reference $\mapsto$ dataset.aut, ARE, $C = 1$
    - r02 … mixture reference $\mapsto$ dataset.mix, ARE, $C = 1$
    - r03 … device reference $\mapsto$ dataset.dev, ARE, $C \geq 1$
    - r04 … geo-location reference $\mapsto$ dataset.loc, ARE, $C = 1$
    - d01 … date and time, seconds since epoch (UTC), ADE, $C = 1$
    - d02 … beaker volume, ADE, $C = 1$
    - d03 … gross weight, measurement result, ADE, $C = 1$
    - d04 … beaker weight, net weight, measurement result, ADE, $C = 1$
    - d05 … specimen weight, calculated value, ADE, $C = 1$
    - d06 … specimen density, calculated value, ADE, $C = 1$
    - a01 … test name, AAE, $C = 1$
    - a02 … operator name, AAE, $C = 1$
    - a03 … procedure description, AAE, $C = 1$
    - a04 … calculation description, formula, AAE, $C = 1$
    - a05 … general description, AAE, $C = 1$
  - **Solid specimen density**
  - s02 … solid specimen density, specimen I, $C = 1$
    - obj … object type descriptor, 'struct_test_ssd1', string, $C = 1$
    - ver … version number [major, minor], unsigned 16bit-integer tuple, $C = 1$
    - r01 … author reference $\mapsto$ dataset.aut, ARE, $C = 1$
    - r02 … specimen reference $\mapsto$ dataset.spm, ARE, $C = 1$
    - r03 … device reference $\mapsto$ dataset.dev, ARE, $C \geq 1$
    - r04 … geo-location reference $\mapsto$ dataset.loc, ARE, $C = 1$
    - d01 … date and time, seconds since epoch (UTC), ADE, $C = 1$
    - d02 … specimen weight, measurement result, ADE, $C = 1$
    - d03 … floating specimen weight, measurement result, ADE, $C = 1$
    - d04 … water temperature of water basin, ADE, $C = 1$
    - d05 … density of water in basin, calculated value, ADE, $C = 1$
    - d06 … weight of displaced water, calculated value, ADE, $C = 1$
    - d07 … specimen volume, calculated value, ADE, $C = 1$
    - d08 … specimen density, calculated value, ADE, $C = 1$
    - a01 … test name, AAE, $C = 1$
    - a02 … operator name, AAE, $C = 1$
    - a03 … procedure description, AAE, $C = 1$
    - a04 … calculation description, formula, AAE, $C = 1$
    - a05 … general description, AAE, $C = 1$
  - s03 … solid specimen density, specimen II, $C = 1$
    - … see substructure dataset.tst.s02
  - **Ultrasonic measuring distance**
  - s04 … ultrasonic measuring distance, specimen I, $C = 1$
    - obj … object type descriptor, 'struct_test_umd1', string, $C = 1$
    - ver … version number [major, minor], unsigned 16bit-integer tuple, $C = 1$
    - r01 … author reference $\mapsto$ dataset.aut, ARE, $C = 1$
    - r02 … specimen reference $\mapsto$ dataset.spm, ARE, $C = 1$
    - r03 … device reference $\mapsto$ dataset.dev, ARE, $C \geq 1$
    - r04 … geo-location reference $\mapsto$ dataset.loc, ARE, $C = 1$
    - d01 … date and time, seconds since epoch (UTC), ADE, $C = 1$
    - d02 … total distance, measurement result, ADE, $C = 1$
    - d03 … thickness of spacer disks, steel washer, measurement result, ADE, $C = 1$
    - d04 … distance between actuator and sensor, calculated value, ADE, $C = 1$
    - a01 … test name, AAE, $C = 1$
    - a02 … operator name, AAE, $C = 1$
    - a03 … procedure description, AAE, $C = 1$
    - a04 … calculation description, formula, AAE, $C = 1$
    - a05 … general description, AAE, $C = 1$
  - s05 … ultrasonic measuring distance, specimen II, $C = 1$
    - … see substructure dataset.tst.s04
  - **Ultrasonic pulse transmission test**
  - s06 … ultrasonic pulse transmission test (compression wave), specimen I, $C = 1$
    - obj … object type descriptor, 'struct_test_utt', string, $C = 1$
    - ver … version number [major, minor], unsigned 16bit-integer tuple, $C = 1$
    - r01 … author reference $\mapsto$ dataset.aut, ARE, $C = 1$
    - r02 … specimen reference $\mapsto$ dataset.spm, ARE, $C = 1$
    - r03 … device reference $\mapsto$ dataset.dev, ARE, $C \geq 1$
    - r04 … geo-location reference $\mapsto$ dataset.loc, ARE, $C = 1$
    - d01 … date and time, seconds since epoch (UTC), ADE, $C = 1$
    - d02 … time elapsed between adding water to cement and test start, ADE, $C = 1$
    - d03 … number of interval steps, number of measurements, ADE, $C = 1$
    - d04 … interval length, time span between measurements, ADE, $C = 1$
    - d05 … device setting, pulse generator voltage, ADE, $C = 1$
    - d06 … device setting, pulse generator pulse width, ADE, $C = 1$
    - d07 … device setting, oscilloscope sampling rate, ADE, $C = 1$
    - d08 … recording block size, number of recorded samples, ADE, $C = 1$
    - d09 … number of initial samples before trigger point, ADE, $C = 1$
    - d10 … number of recorded signals, ADE, $C = 1$
    - d11 … signal/specimen maturity array $[N_{\text{signal}} \times 1]$, ADE, $C = 1$
    - d12 … signal sample time array $[N_{\text{sample}} \times 1]$, ADE, $C = 1$
    - d13 … signal amplitude matrix $[N_{\text{sample}} \times N_{\text{signal}}]$, ADE, $C = 1$
    - a01 … test name, AAE, $C = 1$
    - a02 … operator name, AAE, $C = 1$
    - a03 … procedure description, AAE, $C = 1$
    - a04 … calculation description, formula, AAE, $C = 1$
    - a05 … general description, AAE, $C = 1$
    - a06 … settings file path, full qualified path, AAE, $C = 1$
    - a07 … settings file name, AAE, $C = 1$
    - a08 … settings file hash, sha-256, AAE, $C = 1$
    - a09 … measurements file path, full qualified path, AAE, $C = 1$
    - a10 … measurements file name, AAE, $C = 1$
    - a11 … measurements file hash, sha-256, AAE, $C = 1$
    - a12 … signal data directory path, full qualified path, AAE, $C = 1$
    - a13 … signal data file path list, full qualified paths $[N_{\text{signal}} \times 1]$, AAE, $C = 1$
    - a14 … signal data file name list $[N_{\text{signal}} \times 1]$, AAE, $C = 1$
    - a15 … signal data file hash list, sha-256 $[N_{\text{signal}} \times 1]$, AAE, $C = 1$
  - s07 … ultrasonic pulse transmission test (shear wave), specimen II, $C = 1$
    - … see substructure dataset.tst.s06
  - **Specimen temperature**
  - s08 … specimen temperature, specimen II, $C \geq 0$
    - obj … object type descriptor, 'struct_test_tem', string, $C = 1$
    - ver … version number [major, minor], unsigned 16bit-integer tuple, $C = 1$
    - r01 … author reference $\mapsto$ dataset.aut, ARE, $C = 1$
    - r02 … specimen reference $\mapsto$ dataset.spm, ARE, $C = 1$
    - r03 … device reference $\mapsto$ dataset.dev, ARE, $C \geq 1$
    - r04 … geo-location reference $\mapsto$ dataset.loc, ARE, $C = 1$
    - d01 … date and time, seconds since epoch (UTC), ADE, $C = 1$
    - d02 temperature measurement time array, specimen maturity $[N_{\text{signal}} \times 1]$, ADE, $C = 1$
    - d03 … thermocouple 1, temperature amplitude array $[N_{\text{signal}} \times 1]$, ADE, $C = 1$
    - d04 … thermocouple 2, temperature amplitude array $[N_{\text{signal}} \times 1]$, ADE, $C = 1$
    - d05 … thermocouple 3, temperature amplitude array $[N_{\text{signal}} \times 1]$, ADE, $C = 1$
    - d06 … thermocouple 4, temperature amplitude array $[N_{\text{signal}} \times 1]$, ADE, $C = 1$
    - a01 … test name, AAE, $C = 1$
    - a02 … operator name, AAE, $C = 1$
    - a03 … procedure description, AAE, $C = 1$
    - a04 … calculation description, formula, AAE, $C = 1$
    - a05 … general description, AAE, $C = 1$
    - a06 … placement of thermocouple 1, AAE, $C = 1$
    - a07 … placement of thermocouple 2, AAE, $C = 1$
    - a08 … placement of thermocouple 3, AAE, $C = 1$
    - a09 … placement of thermocouple 4, AAE, $C = 1$
    - a10 … temperature data directory path, full qualified path, AAE, $C = 1$
    - a11 … temperature data file path, AAE, $C = 1$
    - a12 … temperature data file name, AAE, $C = 1$
    - a13 … temperature data file hash, sha-256, AAE, $C = 1$
  - **Environmental conditions**
  - s09 … environment temperature, $C = 1$
    - obj … object type descriptor, 'struct_test_env1', string, $C = 1$
    - ver … version number [major, minor], unsigned 16bit-integer tuple, $C = 1$
    - r01 … author reference $\mapsto$ dataset.aut, ARE, $C = 1$
    - r02 … device reference $\mapsto$ dataset.dev, ARE, $C \geq 1$
    - r03 … geo-location reference $\mapsto$ dataset.loc, ARE, $C = 1$
    - d01 … date and time, seconds since epoch (UTC), ADE, $C = 1$
    - d02 … environment temperature at the beginning of the test run, ADE, $C = 1$
    - a01 … test name, AAE, $C = 1$
    - a02 … operator name, AAE, $C = 1$
    - a03 … procedure description, AAE, $C = 1$
    - a04 … calculation description, formula, AAE, $C = 1$
    - a05 … general description, AAE, $C = 1$

(c)

Atomic Reference Element (ARE)

- ./ … parent structure node
  - obj … object type descriptor, 'ARE', string, $C = 1$
  - ver … version number [major, minor], unsigned 16bit-integer tuple, $C = 1$
  - t … tag, a descriptive name, string, $C = 1$
  - i … referenced id, unsigned 16bit integer, $C = 1$
  - r … referenced structure element, cell array of strings, $C = 1$
  - d … reference description, string, $C = 1$

Atomic Attribute Element (AAE)

- ./ … parent structure node
  - obj … object type descriptor, 'AAE', string, $C = 1$
  - ver … version number [major, minor], unsigned 16bit-integer tuple, $C = 1$
  - t … tag, a descriptive name, string, $C = 1$
  - v … value, string or cell array of strings, $C = 1$
  - d … value description, string, $C = 1$

Atomic Data Element (ADE)

- ./ … parent structure node
  - obj … object type descriptor, 'ADE', string, $C = 1$
  - ver … version number [major, minor], unsigned 16bit-integer tuple, $C = 1$
  - t … tag, a descriptive name, string, $C = 1$
  - vt … value data type, see also predefined value types, $C = 1$
  - v … value (SI units), of type ADE.vt, $C = 1$
  - u … unit, SI unit, string, $C = 1$
  - d … value description, string, $C = 1$

Value types vt	Description
string	character array, character = 8 bit
string_arr	1D cell array of strings (vector)
string_mat	2D cell array of strings (matrix)
boolean	unsigned integer, 8 bit (boolean)
boolean_arr	1D array of type boolean (vector)
boolean_mat	2D array of type boolean (matrix)
uint	unsigned integer (uint)
uint_arr	1D array of type uint (vector)
uint_mat	2D array of type uint (matrix)
int	signed integer (int)
int_arr	1D array of type int (vector)
int_mat	2D array of type int (matrix)
single	single precision floating point number (float)
single_arr	1D array of type float (vector)
single_mat	2D array of type float (matrix)
double	double precision floating point value (double)
double_arr	1D array of type double (vector)
double_mat	2D array of type double (matrix)

Supplementary Figure 1: Tree view of the datasets' hierarchical data structure of test series 1. **(a)** Substructures containing the metadata. **(b)** Test collection substructure. **(c)** Atomic structure elements. Legend: C … cardinality of structure element.

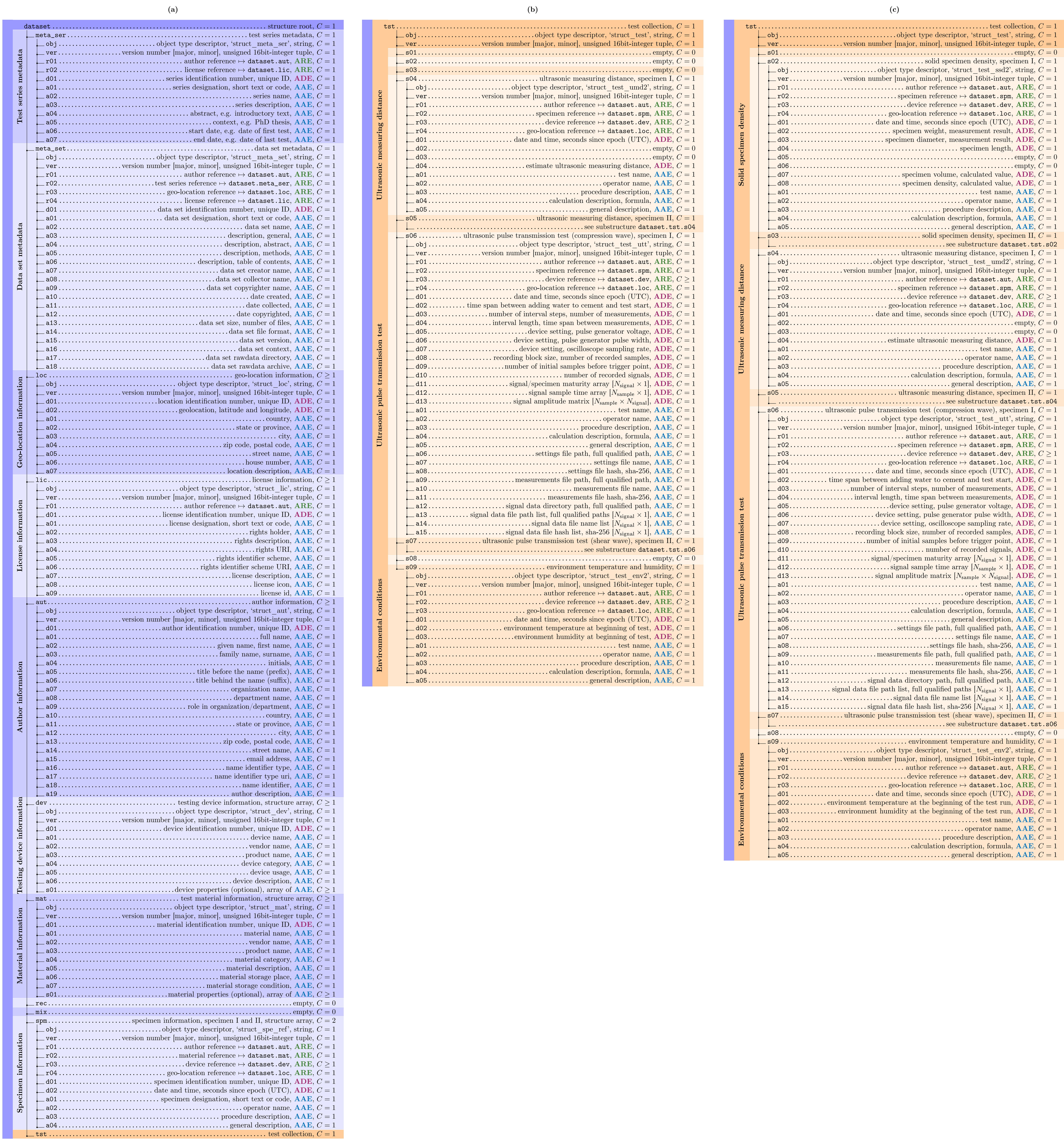

Supplementary Figure 2: Tree view of the datasets' hierarchical data structure in Test series 5 (reference tests on air), Test series 6 (reference tests on water) and Test series 7 (reference tests on aluminium cylinder). **(a)** Substructures containing the metadata – datasets in test series 5, 6, and 7. **(b)** Test collection substructure – datasets in test series 5 and 6. **(c)** Test collection substructure – datasets in test series 7. Note: The tree view and the data type description of the atomic elements AAE, ARE and ADE, please find in Supplementary Figure 1 (c), p. 2. Legend: C … cardinality of structure element.
